# Schema therapy versus treatment as usual for outpatients with difficult-to-treat depression: study protocol for a parallel group randomized clinical trial (DEPRE-ST)

**DOI:** 10.1186/s13063-024-08079-9

**Published:** 2024-04-16

**Authors:** Ida-Marie T. P. Arendt, Matthias Gondan, Sophie Juul, Lene Halling Hastrup, Carsten Hjorthøj, Bo Bach, Poul Videbech, Martin Balslev Jørgensen, Stine Bjerrum Moeller

**Affiliations:** 1 Department of Psychology, University of Southern Denmark, Campusvej 55, 5230 Odense, Denmark; 2 Department of Trauma- and Torture Survivors, Mental Health Services in the Region of Southern Denmark, Vestre Engvej 51, 7100 Vejle, Denmark; 3 Department of Psychology, Universität Innsbruck, Innrain 52, 6020 Innsbruck, Austria; 4 Copenhagen Trial Unit, Centre for Clinical Intervention Research, Copenhagen University Hospital, Rigshospitalet, Blegdamsvej 9, 2100 Copenhagen Ø, Denmark; 5 Research Unit of Stolpegaard Psychotherapy Centre, Mental Health Services, Capital Region of Denmark, Stolpegaardsvej 20, 2820 Gentofte, Denmark; 6 Psychiatric Research Unit, Psychiatry in Region Zealand, Faelledvej 6, 4200 Slagelse, Denmark; 7 Danish Centre for Health Economics (DaCHE), University of Southern Denmark, Campusvej 55, 5230 Odense M, Denmark; 8 Copenhagen Research Center for Mental Health – CORE, Mental Health Services, Capital Region of Denmark, Copenhagen, Denmark; 9 Department of Public Health, Section of Epidemiology, University of Copenhagen, Øster Farimagsgade 5, 1353 Copenhagen K, Denmark; 10 Department of Psychology, University of Copenhagen, Øster Farimagsgade 2a, 1353 Copenhagen K, Denmark; 11 Center for Personality Disorder Research, Mental Health Services in Region Zealand, Fælledvej 6, 4Th Floor, 4200 Slagelse, Denmark; 12 Centre for Neuropsychiatric Depression Research, Nordstjernevej 41, Mental Health Centre Glostrup, 2600 Glostrup, Denmark; 13 Psychiatric Centre Copenhagen, Mental Health Services, Capital Region of Denmark, Frederiksberg Hospital, Hovedvejen 17, 2000 Frederiksberg, Denmark; 14 Department of Clinical Medicine, University of Copenhagen, Blegdamsvej 3B, 2200 Copenhagen N, Denmark

**Keywords:** Schema therapy, Difficult-to-treat depression, Treatment-resistant depression, Treatment refractory depression, Chronic depression, Depressive personality disorder, Persistent depressive disorder, Randomized controlled trial, Psychotherapy, Childhood trauma

## Abstract

**Background:**

About one third of patients with depression are in a condition that can be termed as “difficult-to-treat”. Some evidence suggests that difficult-to-treat depression is associated with a higher frequency of childhood trauma and comorbid personality disorders or accentuated features. However, the condition is understudied, and the effects of psychotherapy for difficult-to-treat depression are currently uncertain. The aim of this trial is to investigate the beneficial and harmful effects of 30 sessions of individual schema therapy versus treatment as usual for difficult-to-treat depression in the Danish secondary, public mental health sector.

**Methods:**

In this randomized, multi-centre, parallel-group, superiority clinical trial, 129 outpatients with difficult-to-treat depression will be randomized (1:1) to 30 sessions of individual schema therapy or treatment as usual; in this context mainly group-based, short-term cognitive behaviour or psychodynamic therapy. The primary outcome is the change from baseline in depressive symptoms 12 months after randomization, measured on the observer-rated 6-item Hamilton Rating Scale for Depression. The secondary outcomes are health-related quality of life assessed with the European Quality of Life 5 Dimensions 5 Level Version, functional impairment assessed with the Work and Social Adjustment Scale, psychological wellbeing assessed with the WHO-5 Well-being Index, and negative effects of treatment assessed with the Negative Effects Questionnaire. Exploratory outcomes are improvement on patient self-defined outcomes, personal recovery, anxiety symptoms, anger reactions, metacognitive beliefs about anger, and perseverative negative thinking. Outcomes will be assessed at 6, 12, and 24 months after randomization; the 12-month time-point being the primary time-point of interest. Outcome assessors performing the depression-rating, data managers, statisticians, the data safety and monitoring committee, and conclusion makers for the outcome article will be blinded to treatment allocation and results. To assess cost-effectiveness of the intervention, a health economic analysis will be performed.

**Discussion:**

This trial will provide evidence on the beneficial and harmful effects, as well as the cost-effectiveness of schema therapy versus treatment as usual for outpatients with difficult-to-treat depression. The results can potentially improve treatment for a large and understudied patient group.

**Trial registration:**

ClinicalTrials.gov NCT05833087. Registered on 15th April 2023 (approved without prompts for revision on 27th April 2023).

**Supplementary Information:**

The online version contains supplementary material available at 10.1186/s13063-024-08079-9.

## Introduction

### Background and rationale {6a}

Major depressive disorder (MDD)—characterized by depressed mood, loss of interest, enjoyment, and energy, and a number of psychosomatic and cognitive symptoms (International Classification of Diseases 10th Revision, ICD-10) [1, 2]—is one of the most common mental disorders, with a life-time prevalence of 15% in Europe [3]. In Denmark, the estimated yearly prevalence of MDD is more than 500,000 patients [4], or just below 10% of the Danish population.

It is estimated that 15–35% of all patients with MDD suffer from Difficult-to-Treat Depression (DTD) [5]. DTD can be defined broadly as “depression that continues to cause significant burden despite usual treatment efforts” [6]. However, there is no consensus of a clearer definition and delineation of DTD [2, 7]. In line with a recent Danish clinical guideline on the subject [8], this trial will pragmatically define DTD as either chronic depression—minimum 2 years of continuous MDD (excluding dysthymia)—or treatment-resistant depression, defined as continuous depression after treatment attempts with minimum two antidepressants of different classes in optimal doses and for sufficient time [5].

A disproportionate part of the societal costs for MDD is probably due to patients with DTD; international studies show them twice as likely to be hospitalized in comparison to non-chronic depression patients, and their general costs for health service use are as much as 40% higher than those for other depression patients [9, 10]. Consequences of DTD for the individual include increased suicidal risk as well as non-suicidal mortality (e.g. increased risk of coronary disease), compared to patients with non-chronic MDD [11, 12]. Lower quality of life and social, emotional, and physical functioning have also been found in comparison to non-DTD patients [13], as well increased burden on relatives [7].

Interestingly, DTD patients seem to differ substantially from non-DTD patients in several aspects of personality development and related functioning: Adverse events in childhood are seen in up to 75% of patients with DTD [14–17], as opposed to about 46% in a patients with depression in general [17]. Further, a higher prevalence of comorbid personality disorders is seen, as well as specific dysfunctional personality features, interpersonal behaviour, and cognitive styles [18, 19]. The inclusion of “Depressive personality disorder” as a proposed distinct personality disorder in the Diagnostic and Statistical Manual of Mental Disorders 4th edition [20] also supports the notion that these patients posit accentuated personality features that may precede, exacerbate, or maintain the condition [21].

### The evidence base for schema therapy

In recent years, the number of randomized clinical trials of schema therapy (ST) has increased. Systematic reviews support the effectiveness of ST for personality disorders, eating disorders, post-traumatic stress disorder, anxiety, and obsessive–compulsive disorder [22–24]. One randomized controlled trial on patients with MDD, of which 67% had chronic depression, compared CBT and ST and found no significant differences on depressive symptoms between the two [25], while another randomized controlled trial on primarily inpatients with severe MDD found no difference in treatment effects between either ST, CBT, or supportive psychotherapy [26]. However, no randomized clinical trials currently exist assessing the effects of schema therapy for DTD. Two multiple case series on chronic depression have been made, showing promising effects [27, 28], but this can only be considered preliminary evidence when it comes to concluding whether ST is a viable alternative to usual treatment.

### Current evidence base for psychotherapy for DTD

To assess the current evidence base for beneficial and harmful effects of psychotherapy for DTD, we searched for systematic reviews and meta-analyses of RCTs assessing any psychotherapy type versus any control intervention for difficult-to-treat depression in PubMed, PsycInfo, and the Cochrane Library, using the following search terms “difficult-to-treat depression”, “chronic depression”, and “treatment resistant depression” in title or abstract. The final search terminated on 31st October 2023 and yielded 421 results. One author (IMTPA) and a student assistant screened all titles and abstracts for relevant systematic reviews. For reviews on pharmacological and combination/augmentation strategies for treatment-resistant depression, please refer to Davies et al. [29] and Scott et al. [30].

We identified six systematic reviews in total (see Additional file 1 for individual characteristics and conclusions of reviews): two systematic reviews with meta-analysis were on chronic depression [31, 32], and four were on treatment-resistant depression [2, 33–35] (all defining treatment resistance as at least one failed attempt at treatment with antidepressant medication). All reviews concluded that there was significant beneficial effect with standardized effect sizes between 0.23 and 0.42 of adding psychotherapy to care as usual (mostly psychopharmacological treatment). No difference was seen in depressive symptoms post-treatment between types of psychotherapy, and only scarce evidence for long-term effects was found. However, the majority of reviews had methodological shortcomings. These included not having pre-registered or published a protocol, lack of Grading of Recommendations, Assessment, Development, and Evaluation (GRADE) assessments [36] and Trial Sequential Analyses [37–40], and most studies not reporting on adverse events in relation to treatment. Therefore, the beneficial and harmful effects of psychotherapy for DTD remain uncertain. Further, it is an unresolved question how the effects of treatment of DTD relate to effects of psychotherapy for MDD in general, which are mostly found to be moderate to large (albeit possibly inflated due to including a wait list as comparison, e.g. *g* = 0.79 for CBT vs control groups), but with overall high risk of bias in most trials [41, 42].

### How the intervention might work

ST was developed out of the CBT depression model for patients not responding well to the “here-and-now” focus of CBT, the use of cognitive and behavioural techniques only, and CBT’s relatively neutral role of the therapist in the therapeutic relationship [43]. ST may be a particularly promising treatment for complex cases with DTD, as it targets both adverse childhood experiences and accentuated characterological features. In the transdiagnostic ST model, experiences of unmet basic emotional needs in childhood create early maladaptive schemas (EMSs), which are latent, stable, trait-like representations about the world, oneself, and the future [44]. A recent meta-analysis showed that EMSs are generally elevated in MDD [45], lending support to the etiological ST model proposed by Renner et al. [44], which hypothesizes that strong EMS are a risk factor for chronic depression.

Going to the “root of problems”, ST could potentially have a better and more enduring effect on the depressive symptoms and other patient-relevant outcomes. However, if ST proves to not have a superior treatment effect to TAU, this would support the conclusions of the earlier mentioned reviews that most psychotherapies do not substantially differ in their effect on DTD.

Regarding cost-effectiveness, the costs of a longer duration of ST therapy, as opposed to the shorter treatment as usual (TAU) psychotherapies (mostly CBT and psychodynamic therapy) currently provided in the Danish mental health care system, could prove to be offset by greater and/or longer-lasting effects. However, if a lack of cost-effectiveness is observed, time, effort, and costs should be put into optimizing the current treatments on offer to enhance treatment effects.

### Objectives {7}

The primary aim of this trial is to investigate the beneficial and harmful effects of schema therapy of up to 30 sessions versus treatment as usual (TAU) for outpatients with DTD in the short and long term.

### Trial design {8}

This is a multi-center, two-arm, parallel-group, assessor-blinded, randomized controlled superiority trial. Participants will be allocated 1:1 to either 30 sessions of ST or to TAU for treating DTD. The first participant was randomized on April 17th, 2023.

## Methods: participants, interventions and outcomes

### Study setting {9}

The treatment will take place at four psychiatric treatment sites in the Southern (Odense) and Capital (Copenhagen/Nørrebro, Frederiksberg, Ballerup) Regions of Denmark, all receiving patients from suburban or urban areas. The clinics involved are outpatient clinics in the secondary mental health care system. The initial screening and assessment will take place at the clinics or via secure online connections. For addresses and contact information for the participating clinics, please see the registration in Clinicaltrial.gov as listed above.

In the case the recruitment flow or other circumstances require so, additional sites may be added after the initiation of recruitment.

### Eligibility criteria {10}

Participant eligibility criteria are as follows:

• Outpatients.
• Aged 18 or above.
• Referred to treatment for depression in a psychiatric clinic, or already in treatment at the clinic and eligible for a second treatment package at the time of inclusion (for an explanation of treatment packages, see Item 11a: Intervention description).
• Meeting the diagnosis of chronic or treatment-resistant depression as follows:

Clinical MDD as assessed by the M.I.N.I.-5 diagnostic interview [46]; see further under item 18a): MDD duration minimum 2 years OR persistent MDD after ≥ 2 trials of antidepressants from different classes, in an adequate dosage and time period (≥ 4 weeks) OR moderate treatment resistance as measured on the Maudsley Staging Model (MSM), score > 6.

• Minimum a score of 9 points on the 6 item Hamilton Rating Scale for Depression (HAMD-6), corresponding to moderate to severe MDD.
- Exclusion criteria are as follows:
• Alcohol or substance abuse.
• Bipolar disorder.
• Psychotic disorders or current psychotic symptoms of a character that precludes treatment in a depression treatment package.
• Acute suicidal risk.
• Mental disability (estimated IQ < 70).
• Non-Danish speaker, to the extent that the person cannot read and respond to the self-reported outcome questionnaires in Danish.
• Having received treatment with ST in the past 5 years.
• Pregnancy known at the time of inclusion (since childbirth and infant care could lead to difficulties in upkeeping continuous treatment appointments).

Comorbid mental disorders other than MDD are not exclusion criteria, unless it is deemed necessary that the patient is redirected to a treatment primarily focused on another mental disorder than MDD.

#### Treatment site and therapist eligibility

The treatment sites are selected based on their willingness to participate. All sites are outpatient clinics offering standardized treatment for MDD, serving as TAU in the trial. ST-therapists are authorized psychologists, social workers, or nurses with a background of providing CBT, who will be recruited on the basis of willingness to participate and training availability. Approximately one third of these therapists have prior ST-training and have practiced ST for several years.

The therapists who have no prior ST experience will complete a 4-day workshop including practical exercises, introducing the ST model. After a period with opportunity to become practically familiar with the ST model, all therapists, regardless of prior experience, will complete an additional 4 days of training with focus on treating patients with DTD with ST. The training will focus on learning the ST-techniques through practical exercises.

In the case of dropout of ST-therapists, new eligible therapists will be given about a week of ST-education and training and be called to participate in a half-day workshop with education and training specifically on the manual for this trial.

### Who will take informed consent? {26a}

Informed consent from the participant will be obtained as part of the recruitment process which is undertaken by the Principal Investigator (an experienced, authorized clinical psychologist and PhD-student) and a research assistant (a master level psychology student, supervised by the Principal Investigator). Potential participants are first contacted and given brief oral information about the trial as well as detailed written information as approved by the Regional Ethics Committee (see Additional file 2). After given a minimum of 24 h to consider participation, and if the participant is still interested, they are invited for a physical or online recruitment and baseline assessment session. The baseline assessment takes place in a quiet setting, either with physical presence in an office at a research or treatment site or if preferred by the participant, online on a secure platform, in which case the participant will be asked to ensure that the interview can take place privately and uninterrupted. This session starts with an additional summary and dialogue about the course of the trial and what it entails for the participant. The participants will be informed that participation is voluntary and that withdrawal from the trial is possible at any time without implications for their possibilities to receive psychiatric treatment.

Informed consent for participation is then obtained in a paper or pdf consent sheet (for consent form, written and verbal information, please see Additional file 3).

### Additional consent provisions for collection and use of participant data and biological specimens {26b}

An ancillary, mixed methods study investigating the patient perspective on good treatment outcome will be conducted. This study will collect primarily qualitative data but will also use some quantitative data which is collected as part of the main trial (The Psychological Outcome Profiles (PSYCHLOPS) questionnaire, as described below). A separate consent procedure, including participant material and consent form, will be presented.

Supplementary studies on patient and clinician treatment experiences are currently being considered. Should these be initiated, they too will require a separate consent form and participation material.

## Interventions

### Explanation for the choice of comparators {6b}

This trial compares the effect of ST with TAU. TAU is chosen as a comparator intervention in order to be able to discern the effect of a long-term, individual treatment with ST in comparison to the current practice for psychotherapeutic treatment for DTD in the Danish secondary mental health system.

### Intervention description {11a}

The participants will be randomized 1:1 to receive either up to 30 weekly, 45–60-min individual sessions of ST during a period of approximately 10 months (allowing for sick days and holidays), or TAU. All participants in the ST arm will additionally be offered pharmacological treatment, meetings with next-of kin, etc., equal to that offered in the TAU arm (as described below).

A treatment manual for ST has been developed for the trial. The manual prescribes treatment in four phases with a progression from conceptualization and exploration of schemas and modes, through a focus on working through childhood experiences, then a focus on helping patients to strengthen their “Healthy Adult Mode” and letting that mode take the lead in their current lives, and concluding in a phase preparing for end-of-treatment and relapse prevention. Since ST is tailored to the unique schemas, modes, and emotional needs of each patient, the manual functions less as a session-by-session script, but rather as a principle-driven compendium of intervention strategies outlining key schema therapy concepts for various phases. It is stressed that experiential interventions (such as chair work or imagery) should be incorporated in at least every second session.

Regarding the TAU, all Danish patients with moderate to severe MDD who are deemed suitable for out-patient psychiatric treatment are officially treated according to standard “treatment packages” which consists of preparatory and diagnostic sessions, psychotherapy (in group or individual), prescription and monitoring of psychopharmacological treatment by a psychiatrist, as well as meetings with the participation of next-of-kin and/or collaboration partners in other public instances [47]. However, the extent of the different elements in a treatment package varies somewhat among the participating psychiatric sites, giving a certain heterogeneity to the TAU-condition: Three out of four sites primarily offer Cognitive Behavioural Therapy (CBT), while one site offers both CBT and psychodynamic therapy. One site offers only individual therapy. The three remaining sites offer mostly group therapy (all according to similar, but locally adapted manuals), lasting from 90 min and 16 sessions (psychodynamic group; one site) to 120 or 150 min in 13–14 sessions (CBT groups; three sites). At these sites, individual psychotherapy is offered to a smaller subgroup of patients from 6 sessions up to 16 sessions or more (varying by site). The type and number of sessions for psychotherapeutic TAU treatments will be registered for each participant. Psychopharmacological treatment and changes in medicine prescriptions are permitted if prescribed by the psychiatrists working at the treatment sites, following the usual pharmacological treatment methods. Medication use will be monitored via the electronic patient records.

The schema therapists, who are all employed as regular mental health practitioners at the sites in the trial, will simultaneously function as TAU-therapists along with other TAU-therapists employed at their respective sites. The ST-therapists will be given strict instructions not to utilize ST concepts or interventions in TAU.

### Criteria for discontinuing or modifying allocated interventions {11b}

Discontinuation of the intervention can happen based on the following:

- Professional evaluation from both the participant’s therapist and the participant, based on substantial and lasting improvement of the participant’s condition, or conversely, lack of effect or adverse effects of treatment, low participant motivation, or adherence to treatment.
- Explicit (verbalized) wish to terminate treatment from the participant, or continuous no-show, as specified by the individual site’s guidelines for treatment termination (e.g. after two–three consecutive no-shows without prior cancellation).
- The emergence of exclusion criteria that were not present or known at the time of inclusion (i.e. bipolar disorder, psychotic symptoms, substantial and continuous substance abuse) when these lead to the participant necessarily being referred for treatment for anything other than MDD.

### Modification of treatment can happen as follows

- If a participant in individual therapy becomes pregnant, has to move away or for another reason needs to finish treatment earlier than planned, it will be attempted to finish the remaining sessions of treatment, e.g. by a higher frequency of sessions until the participant’s due date.
- In ST, the therapy can be paused and exchanged for, e.g. psychiatric hospitalization or supportive counselling, if the participant’s condition has worsened to a degree where it is deemed not possible or unethical to continue ST. If the participant’s condition subsequently stabilizes, ST can be resumed; if not, the participant will be considered a treatment dropout.
- Additional treatments, e.g. exercise groups, Pulsed Electromagnetic Field Therapy, Electroconvulsive Therapy, are in general not allowed in the ST-arm. Exceptions are made on an individual basis in the case of serious worsening of the participant’s condition where it is deemed unethical to not offer the additional treatment to the participant.
- In TAU, there are no restrictions to treatment modification or additional treatment offers.

### Strategies to improve adherence to interventions {11c}

After training in ST, the therapists’ competency and adherence to the ST protocol will be assessed post-training via video recordings of a therapy session with a patient not involved in the study intervention. Informed consent will be obtained for this purpose. Two trial investigators will review and rate these videos, provide feedback, and any therapist not meeting the competency standards will be required to submit new recordings until satisfactory performance is achieved.

After the completement of training and throughout the treatment phase of the trial, therapists will receive 1.5 h of monthly, online group supervision.

One third of all ST sessions in the trial will be video-recorded and a selection of these for each therapist will be evaluated for general competency and adherence using structured rating instruments.

### Relevant concomitant care permitted or prohibited during the trial {11d}

Concomitant psychopharmacological or psychotherapeutic treatment outside of the treatment sites is generally discouraged when under treatment in the secondary mental health sector. However, should the participant seek out external treatment, the participant will not be excluded from the trial, and the extent of this treatment will be monitored.

For the ST-arm, it will be attempted to restrict treatment to ST, psychopharmacological treatment, and 1–2 sessions with network or next-to-kin as needed. In the (expected rare) case of severe deterioration of a participant’s condition where the already extended treatment is not deemed sufficient, additional treatment as available within the treatment options of the respective site can be offered; this will be decided on a case-to-case basis, as described in Item 11b. There is no restriction on the treatment in the TAU-arm given within the sites’ treatment options.

### Provisions for post-trial care {30}

When the participant has terminated or dropped out of treatment, further care can be provided in the primary sector through the participant’s general practitioner.

Additional treatment packages in the TAU-arm may be provided in special cases where it is deemed necessary or beneficial for the participant.

As all treatment takes part in a regional, public psychiatric hospital setting, participants are covered by the public patient insurance in the case of insufficient or harmful treatment or adverse events directly related to treatment [48].

### Outcomes {12}

Measurement time points are at 6, 12, and 24 months, and, conditional on positive results and additional funding, at 36 months after baseline assessments for all outcomes except when stated below (see Table 1). The primary timepoint of interest will be at 12 months after randomization for all outcomes, except for the Psychological Outcomes Profile (PSYCHLOPS), and the European Quality of Life 5 Dimensions 5 Level Version (EQ-5D-5L), as stated below.

**Table 1:**
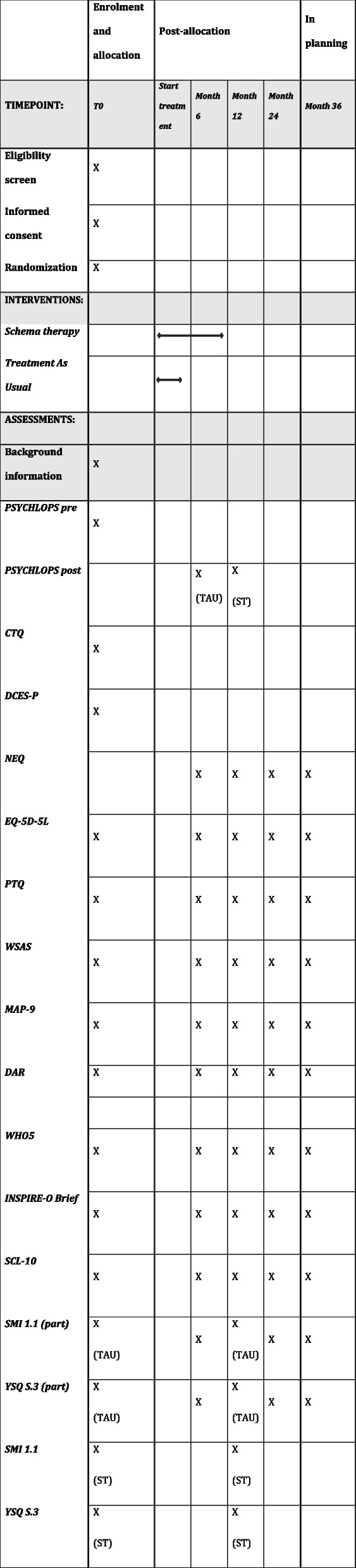
Timeline for assessments

*TAU* Treatment As Usual, *ST* Schema Therapy, *EQ-5D-5L* European Quality of Life 5 Dimensions 5 Level Version, *PSYCHLOPS pre* Psychological outcome profiles pre-therapy, * PSYCHLOPS post* Psychological outcome profiles post-therapy, *CTQ* Childhood Trauma Questionnaire Short Form, *PTQ* The Perseverative Thinking Questionnaire, *WSAS* Work and Social Adjustment Scale, *NEQ* The Negative Effects Questionnaire, * MAP-9* The Metacognitive Anger Processing (MAP) Scale (9 items), *DAR * Dimensions of Anger Reactions Scale Revised, *DCES-P* Depression Change Expectancy Scale—Pessimistic scale, *WHO-5* The WHO-5 Well-Being Index,* SCL-10* The Symptom Checklist (5 items), *SMI 1.1 part* Schema Mode Inventory version 1.1 (Healthy Adult, Vulnerable Child, Punitive and Demanding Parent-modes only), *YSQ S.3 part* Young Schema Questionnaire S-3 (Emotional Deprivation, Abandonment, Mistrust/Abuse, Social Isolation, and Defectiveness schemas only), * SMI 1.1* Schema Mode Inventory version 1.1 (full version), *YSQ S.3* Young Schema Questionnaire S-3 (full version)

#### Primary outcome

The primary outcome will be the change from baseline in severity of depression on the observer-rated 6-item Hamilton Rating Scale for Depression (HAMD-6) [49], assessed by allocation-blinded investigators at 12 months after the baseline assessment, i.e. approximately at the end of treatment for the ST group.

#### Secondary outcomes

All secondary and exploratory outcomes are reported as changes from baseline unless otherwise stated.

The observer-rated HAMD-6 is a secondary outcome at 6 and 24 months.

### Quality of life assessed with the European Quality of Life 5 Dimensions 5 Level Version (EQ-5D-5L)

Quality of life is assessed with the European Quality of Life 5 Dimensions 5 Level Version (EQ-5D-5L), which is a 6-item measure covering 5 domains of health-related quality of life on a five-point scale, as well as an overall health visual analogue scale (VAS) measure. It is often used in cost-effectiveness studies in Denmark as well as internationally and has shown good psychometric properties [50]. Danish norms for the EQ-5D-5L have been calculated [51]. The primary time point of interest will be at 24 months after baseline measurements.

### Level of functioning assessed with the Work and Social Adjustment Scale (WSAS)

This is a 5-item questionnaire reporting subjective functional impairment in work and home managing, social and private leisure activities and functioning in relationships, measured on a Likert-scale from 1 to 8 [52]. The scale was chosen for this trial among several other potential scales measuring functional impairment, since it relates the experienced impairment directly to the patient’s current disorder, thus leaving out impairment due to comorbid somatic illnesses or other problems not directly modifiable by psychotherapy. WSAS exhibits good psychometric properties in clinical samples, including adequate internal consistency, convergence with disorder severity, and sensitivity to treatment-related change. Also, it has good clinical validity and high correlations between clinician and patient reports [52].

### Psychological well-being assessed with the WHO-5 Well-Being Index (WHO-5)

This 5-item questionnaire is one of the most widely used measures to assess psychological well-being, and it is often used in research on depression. Five different aspects of well-being are assessed on a 6-point Likert scale (0–5), reflecting how much of the time they are present. It has excellent psychometric properties with unidimensionality, high construct as well as clinical validity, and acceptable sensitivity and specificity [53].

### Negative effects of treatment assessed with the Negative Effects Questionnaire (NEQ)

The NEQ is a 20-item self-report questionnaire, covering five domains: Symptoms, quality of treatment, dependency, stigma, and hopelessness. The respondent rates any experienced negative effects on a 5-point Likert scale and states whether the negative effect was due to the treatment they received or other circumstances. Negative effects of the treatment will be registered at all measurement time points, since it is important to establish if they are enduring or only transient, such as the experiencing of negative feelings during treatment [54]. The NEQ exhibits good psychometric properties, demonstrating fairness in testing across socioeconomic status in the 0–4 point scale which advances monotonically. It is deemed suitable to monitor effects on an item-level and demonstrates an acceptable person goodness-of-fit [55].

Outcome on the NEQ will be measured as mean negative effect per time point.

#### Exploratory outcomes

##### Personal recovery assessed with the Brief INSPIRE-O

This 5-item questionnaire captures important elements of personal recovery from mental illness: connectiveness, hope, identity, meaning and empowerment (CHIME-model) [56]. Adapted from the original Brief INSPIRE [56], which measures these domains in relation to experiences with the treatment provider (e.g. “My worker helps me to feel supported by other people”), the wordings were changed slightly to instead reflect individual experiences of the recovery process (e.g. “I feel supported by other people”) [57]. The Brief INSPIRE-O was found to have acceptable test–retest reliability, scalability, internal consistency, convergent validity, and sensitivity to change in a Danish psychiatric sample [(Moeller SB, Larsen PV, Austin SF, Slade M, Arendt I-MTP, Simonsen S: Scalability, test-retest reliability and validity of the Brief INSPIRE-O measure of personal recovery in psychiatric services, unpublished), (Moeller SB, Larsen PV, Austin SF, Slade M, Arendt I-MTP, Kring L, et. al.: Sensitivity to change and clinical cutoff of the Brief INSPIRE-O measure of personal recovery in mental health services, unpublished)], as well as satisfactory internal consistency and support for unidimensionality [58].

##### Changes in patient-elected problems assessed with the Psychological Outcome Profiles (PSYCHLOPS)

This self-report questionnaire evaluates changes in 1–2 patient-defined problems and related difficulties on a 6-point Likert Scale [59]. It exists in three versions for use pre-treatment, during, and at the end of treatment. The “pre-” and “end of treatment (post)” versions will be used in this trial and distributed approximately at the end of TAU (6 months after baseline measurements) and at the end of ST (12 months after baseline measurements) for the respective patients in the two arms. PSYCHLOPS has shown an acceptable pre-treatment internal reliability of 0.75 (Cronbach’s alpha) [60], a convergent validity compared to the CORE Outcome Measure (CORE-OM) with a correlation of 0.74 after treatment and a larger effect size than both CORE-OM and HADS (Hospital Anxiety Depression Scale) [60, 61]. This measure is the only of the elected measures for this trial which was not already translated to Danish. It has therefore been translated and back-translated, according to the usual scientific standards, for the use in this trial.

##### Reactions to anger assessed with the Dimensions of Anger Reactions – Revised (DAR)

The 7-item self-report questionnaire, measuring the individual's reactions to experiencing anger, has been found to have good psychometric properties in both clinical and non-clinical populations [62].

##### Anger processing assessed with the Metacognitive Anger Processing scale, Short Version (MAP-9)

This is a 9-item self-report scale, originating from the 26-item Metacognitive Anger Processing scale. It is intended for clinical use for the measurement of anger processing in populations with anger and/or aggression problems. It has shown good psychometric properties in both its long and short versions [63].

##### Repetitive negative thinking assessed with the Perseverative Thinking Questionnaire (PTQ)

Measuring repetitive, negative thinking (RNT) across mental disorders [64], its 15 items evaluate: (a) the core characteristics of RNT, identified as the repetitiveness of RNT, the intrusiveness of RNT, and the difficulty of disengaging from RNT (PTQ-core with 9 items), (b) the perceived unproductiveness of RNT (PTQ-unproductiveness with 3 items), and (c) RNT capturing of mental resources (PTQ-mental capacity with 3 items). The response to each item is given using a 5-point Likert scale from 0 (never) to 4 (almost always), where a higher score reflects a higher level of repetitive negative thinking. A validation article on a Danish clinical population shows good psychometric properties, including good internal consistency and sensitivity to change [65].

##### Anxiety symptoms assessed with the Symptom Checklist-10 (SCL-10)

A self-report, 10-item measure for symptoms of depression and anxiety, the SCL-10 has shown good psychometric properties in a Danish, clinical population [66]. For this trial, only the five items for anxiety will be used.

##### Response and remission of depressive symptoms

Response and remission of depressive symptoms will also be reported as exploratory outcomes. Dichotomized versions of the primary outcome will be used to report the proportions of participants that attained “response”, defined as a 50% reduction in depression symptomatology, and “remission”, defined as absence of illness, i.e. < 5 on the HAMD-6 [67].

#### Mediators

The following mediators and predictors are predefined and planned to be analysed and presented in additional publications:

##### Schema modes assessed with the Schema Mode Inventory 1.1. (SMI) [68]

The ST model contains 14 schema modes which are measured with 118 items. A Danish translation replicated the 14-factor structure of the original SMI with adequate internal consistency and construct validity [69]. To reduce the item load on participants, the SMI in its entirety will only be distributed (twice; at randomization and at 12 months after baseline) to participants who are randomized to ST as it is directly used in ST-treatment. However, all participants will be distributed a subset of the SMI at all time points, measuring Vulnerable Child mode (10 items), Healthy Adult mode (10 items), Demanding Parent (7 items), and Punitive Parent modes (10 items).

##### Early maladaptive schemas assessed with the Young Schema Questionnaire 3 Short Form (YSQ-S3)

YSQ-S3 is a 90-item questionnaire measuring all 18 Early Maladaptive Schemas (EMSs). It was validated in a Danish clinical sample and showed good clinical properties [70]. Again, to reduce the item load on participants, the whole YSQ-S3 will only be distributed (twice; at randomization and at 12 months after baseline) to participants who are randomized to ST, as it is directly used in treatment. However, all participants will be distributed a subset of the YSQ-S3 at all time points: Emotional Deprivation (5 items), Abandonment (5 items), Mistrust/Abuse (5 items), Social Isolation (5 items), and Defectiveness (5 items).

#### Predictors

##### Depression treatment

Data regarding treatment for MDD in earlier and current depressive episodes will be collected, including psychopharmacological treatment, psychotherapeutic treatment, ECT, and psychiatric hospitalization. This information will be entered into the Maudsley Staging Model (MSM) [71] to produce a total treatment resistance score (except for psychotherapeutic treatment, as it is not part of the MSM).

##### Childhood trauma assessed with the Childhood Trauma Questionnaire Short Form (CTQ-SF)

The CTQ-SF consists of 28 items (also including three validity items), measuring five forms of maltreatment—emotional, physical, and sexual abuse, as well as emotional and physical neglect [72]. A Danish translation of the CTQ-SF was validated in a Danish clinical sample, demonstrating acceptable internal consistency as well as convergent validity [73].

##### Expectancies for depressive symptom change assessed with the Depression Change Expectancy Scale-Pessimistic (DCES-P)

Eleven items measure expectations of treatment effects in patients with depression, formulated in pessimistically worded statements. These items were derived from the full DCES which also contains optimistically worded items, which again was adapted from the well-validated Anxiety Change Expectancy scale [74]. The response is made on a 5-point Likert scale from 1 (*strongly agree*) to 5 (*strongly disagree*). The DCES has been validated on a dysphoric student sample and a clinical sample [75].

### Participant timeline {13}

Participants are contacted immediately after identification and referral from the clinic. The intake interview, baseline assessments, and randomization will then take place as soon as possible at the participant’s convenience (most often within less than a week).

After randomization, treatment will commence as soon as a therapist is available. Due to the therapists’ other obligations at the clinic, a waiting time of up to 2 months is pragmatically allowed.

See Table 1 for timeline for assessments.

### Sample size {14}

Sample size planning is based on previous studies that used the short form of the Hamilton Rating Scale for Depression (HAMD-6) as their primary or secondary outcome. In previous studies, the standard deviation of HAMD-6 scores at end-of-treatment was around 3.5 within the intervention arms ( [49, 76]; for a review, see [77]). A difference of 2 units on the HAMD-6 (i.e. *d* = 0.57) is considered clinically relevant [78]; this is the difference we would not like to miss in the comparison of the group averages at the 12-month measurement point. On the HAMD-6 scale (range 0…22), 2 units correspond to an improvement on two of the six items (depressed mood, guilt feelings, work and interest, psychomotor retardation, psychic anxiety, general somatic symptoms). At the conventional significance level of α = 0.05 two-tailed, a total of *N* = 100 participants need to be randomized to detect the relevant group difference with 80% power (as calculated in the software program G*power3 [79]).

The number of randomized participants is increased to account for clustering and dropout: ST is administered individually (not in groups); therefore, cluster effects are expected to be low (intra-cluster correlation = 0.01), but not zero because several participants are treated by the same therapist. Dropout is assumed to be substantial in this patient population and is compensated in the sample size calculation even if the main analysis uses imputation of missing data. With a cluster size of around 5 participants per therapist, and accounting for a dropout of approximately 20%, the total sample size is increased to a total of 129 participants, randomized 1:1 in each intervention arm.

### Recruitment {15}

Participants are recruited as follows: When referred to psychiatric treatment in the Mental Health Services, Capital and Southern Regions of Denmark, all new patients routinely go through an initial assessment interview where the target diagnosis for treatment is established. At this interview, the site’s clinicians also evaluate the patient’s eligibility for the trial. Eligible patients can already here be briefly informed about the trial, given a pamphlet describing the outlines of the trial, and asked for consent to pass on name and phone number to the research team.

All patients are then discussed at a weekly clinical conference, in which it will be decided whether the patient is offered treatment at the site. Here, eligible patients that were not yet introduced to the trial at the intake interview are identified and subsequently contacted. Additionally, patients who have received treatment at the services without sufficient response are also identified and discussed at clinical conferences for referral to the trial in connection with further treatment.

The research staff will be present at clinical conferences at two of the participating sites, while in another site, the recruited schema therapists will identify potential participants. At the final site, the head of clinic will refer the eligible participants directly to the research team.

Approximately 1300 patients yearly initiate treatment with MDD in the three participating sites in the Mental Health Services, Capital Region of Denmark, alone. Additionally, about 700 patients receive a second treatment attempt. If, conservatively estimated, 15% of these have DTD, and 50% of these patients agree to participate, this would give 150 participants from the Capital Region alone in 1 year. With an additional site in Odense in the Southern Region of Denmark, a sufficient flow of participants for the trial can be assumed.

The recruitment period will last approximately 27 months, with a possibility for extension if necessary to meet the planned sample size. Recruitment will be monitored continuously, and bimonthly meetings will be held with the heads of clinic to follow-up on recruitment status and barriers.

## Assignment of interventions: allocation

### Sequence generation {16a}

The allocation sequence, consisting of permuted blocks of various sizes, is generated by Sealed Envelope Ltd [80] (see Additional file 4). The randomization list is then implemented in REDCap and set up for randomization by an external data manager employed at the research support function OPEN in the Region of Southern Denmark. This person does not otherwise undertake data collection or has other direct involvement in the trial.

### Concealment mechanism {16b}

The randomization and allocation to intervention will be executed in REDCap by the research team member after all the baseline measurements have been undertaken. The allocation sequence is in the possession of this data manager until all data has been collected, keeping details about restrictions to randomization concealed from the research team members performing randomization. The allocation sequence will not be visible or directly accessible in REDCap, and it is not possible to influence or change the randomization result.

### Implementation {16c}

Following screening and baseline assessment, participants are randomized to either ST or TAU with a 1:1 allocation. The randomization was initially stratified by depression severity (moderate or severe; HAMD-6) and childhood maltreatment (Childhood Trauma Questionnaire (CTQ-SF), dichotomized) at baseline for the first 14 included participants. It was then, however, identified as an error in the trial design that there was no stratification for treatment site, and this was then applied (instead of stratifying for depression severity and childhood maltreatment) for the remainder of the included participants.

The data management system REDCap (Research Electronic Data Capture), a secure web application for building and managing online surveys and databases, executes randomization.

The research team member performing the screening for inclusion criteria and baseline measurements will inform the participant of his/her intervention allocation once all baseline measurements are completed. The research team member will also inform the relevant clinic and the treating clinician of the results of randomization and submit the YSQ-S3 and SMI for the clinician for use in ST treatment.

## Assignment of interventions: Blinding

### Who will be blinded {17a}

The following trial personnel will be blinded for treatment [81]:

- Outcome assessors (for the observer-rated HAMD-6)
- Data managers
- Statistician
- Data safety monitoring committee (can be unblinded if necessary in relation to patient safety)
- Conclusion makers executing overall conclusions in the outcome article

All pre-treatment measures are taken before randomization, with the exception of the SMI and YSQ-S3, which differ according to allocation (parts of or the whole questionnaire). However, the participant who is administered the self-report measures remains blinded to allocation up until the point where they have completed all baseline questionnaires. Further, as the self-report measures are self-explanatory, the interaction between the researcher and participant is minimal at this part of the assessment, leaving the possibility for influence on the participant’s baseline responses on the SMI and YSQ-S3 improbable.

As researchers carrying out pre-treatment assessments will also subsequently be performing the randomization and treatment allocation as described above, they will become unblinded in relation to making outcome assessments. Other research assistants will therefore be trained to conduct the outcome assessments at post-treatment and follow-up. All outcome assessors will thus be blinded to the assignment of interventions, and central information about the trial design and interventions is withheld from them until the post-treatment and follow-up assessment phases are completed. Outcome data will be entered into REDCap, which will be set up for the outcome assessor so that the allocation of the participant is hidden from view. The research team has no contact with the participants other than that related to assessments.

Given the nature of psychotherapy, including necessary extended training and knowledge acquisition, it is not possible to blind the therapists providing the treatment. Participants can also not be blinded in this trial, given the differentiating nature, format and length of ST and TAU, respectively. Participants will repeatedly be instructed to keep their treatment allocation concealed for the outcome assessors. In case a participant unintentionally reveals their treatment allocation, the outcome assessment will be interrupted immediately, and a different outcome assessor will take over the remaining of the current and future assessments.

A detailed statistical analysis plan is planned to be published before unblinding the data. The data analyst will only be given access to blinded data, i.e. labelled “treatment group A” and “treatment group B” and will perform the statistical analyses as planned before unbreaking of the blind.

Regarding the conclusions of the trial, the trial steering committee will receive blinded statistical analyses with the treatments coded as “A” and “B”. The steering committee will then approve two versions of the abstract, one where it is assumed that schema therapy is Treatment “A”, and one where it is assumed to be Treatment “B”, before the blind is broken [81].

### Procedure for unblinding if needed {17b}

The person performing the inclusion procedures and randomization is not blinded to treatment allocation (after the baseline measurements have been carried out), meaning that any emergency actions where knowledge of treatment allocation is necessary can be performed without the unblinding of the outcome assessors, data analyst, or other relevant parties.

## Data collection and management

### Plans for assessment and collection of outcomes {18a}

For a list of outcomes, please see item 12. All outcomes are attached in Additional file 5, except for forms with copyright (CTQ-SF, SCL-10, SMI, and YSQ-S3).

All observed rated outcomes are entered directly into the REDCap system (see explanation in item 19; Data management), and all patient-reported outcomes are distributed through the REDCap system, and either displayed on a tablet (if the assessment in taking place in person) or on the participant’s own device (in case the assessment is made online).

Information about the participant’s employment, civil status, individual and household income, and education status will also be collected at baseline.

### Training of assessors

The Principal Investigator (the first author of this paper), an experienced clinical psychologist, will perform some of the baseline measurements and train the baseline and outcome assessors in the collection of data. The other assessors are primarily Masters students in Psychology.

A particular emphasis will be put on acquiring skills for conducting the observer rated HAMD-6. Prior to the training of outcome assessors, the Principal Investigator will have attended bi-weekly training assessments at a psychiatric site not in this trial over the course of 1 year, conducting the HAMD-6 on patients with suspected MDD.

Subsequently, the other assessors will witness training videos and live training assessments of the HAMD-6 as described above, as well as conduct a number of HAMD-6 ratings on participants in the trial, supervised by the Principal Investigator.

All HAMD-6 ratings will be audiotaped, and a subsample of these will be used to establish interrater reliability among all raters.

For the M.I.N.I. interview, which is conducted for the collection of baseline data as well as establishment of the presence of some exclusion criteria, the baseline assessor is trained on in vivo patients by the Principal Investigator until a certain level of familiarity and fluency using the instrument has been obtained. The baseline assessor will receive continuous supervision and guidance from the Principal Investigator. The Principal Investigator will make final decisions regarding inclusion or exclusion.

### Plans to promote participant retention and complete follow-up {18b}

At the 6-, 12-, and 24-month time points after baseline measurements, the participants will be contacted by phone, text message, and/or secure e-mail and asked to participate in follow-up assessments. Data will be attempted collected also from participants who have withdrawn from treatment. At the baseline interview, and also in case of dropout of treatment, it is stressed that the participant will still be called for outcome assessments regardless of treatment adherence.

Participants who choose to not commence treatment after randomization, or participants who drop out of treatment, will be contacted by the researchers to collect information about potential causes, and these will be recorded and presented in the resulting outcome article.

Trial participants who express doubts about treatment or are considering to drop out will be invited by their therapist to a collaborative and motivational inquiry about their doubts. Trial participants who are absent from therapy sessions without notice will be contacted for a similar inquiry.

In the bi-monthly status meetings with the heads of clinic, researchers will monitor the inclusion, adherence, and retention of participants as well as therapists and take appropriate action when necessary. Also, continuous communication and yearly meetings are planned with the trial therapists, along with the monthly group supervision as described above, to increase motivation and sense of commitment to the trial.

### Data management {19}

The REDCap system has inbuilt options to verify range and value checks, as well as warnings in the case of missing entries of data points. REDCap is a browser-based platform which requires an individual user profile and password, to be changed at regular intervals. All other data are stored in the OPEN storage directory, a secured data storage platform under the Region of Southern Denmark. Data will be kept until 5 years after the data collection has been completed, which is in accordance with Danish data security laws.

### Confidentiality {27}

All data about potential and enrolled participants are collected and stored in the REDCap database. They will only be passed on, in anonymized form via a secure VPN connection, to the statistician on the project, and to the members of the data safety monitoring committee.

Communication between site representants and research team about participants will be over the phone or on a secure email, and all emails with personal identifying information will be deleted after a maximum of 30 days. This also includes cases where information about a potential or enrolled participant’s health is deemed necessary to pass on to the treatment staff responsible for the participant.

Written consent forms will be kept either electronically in the OPEN Storage directory, or in paper format in locked file cabinets with limited access at one of the trial sites.

Plans for collection, laboratory evaluation, and storage of biological specimens for genetic or molecular analysis in this trial/future use {33}

Not applicable, since no biological specimen data are collected.

## Statistical methods

### Statistical methods for primary and secondary outcomes {20a}

The statistical methods will only be presented here in brief, as they will be published in further detail in a separate statistical analysis plan (SAP). The SAP will be submitted for publication in due time before the end of the data collection.

#### Efficacy analyses

The primary outcome (HAMD-6) is treated as an interval-scaled, normally distributed variable. The efficacy of the therapies will be compared using a multilevel linear regression with therapy arm (levels ST, TAU) as the main effect of interest, center (categorical), and baseline depression symptom level (continuous) as covariates, and therapist as a random factor. The therapy effect will be presented as the covariate-adjusted difference between the change scores in the two therapy arms, along with its 95% confidence interval. With adjustment for baseline severity, this estimate is numerically identical to the comparison of the raw outcomes at end-of-therapy [82].

The primary analysis is based on the full analysis set, with the therapy assigned by randomization. Sensitivity analyses will be carried out for the subset of per-protocol participants with available outcome data. Further sensitivity analyses will be carried out using non-linear regression models (e.g. negative binomial regression) to rule out bias due to ceiling or floor effects in the interval-scaled outcomes. Secondary outcomes will be analysed in a similar way, using generalized linear models depending on the type of the outcome (e.g. multilevel logistic regression for the response rates).

#### Health economic evaluation

To inform policy makers on the cost-effectiveness of the ST intervention, an economic analysis will be carried out. The objective of the analysis is to assess the relative cost-effectiveness of the intervention in comparison with TAU.

The evaluation will adopt a societal perspective when considering the costs associated with the intervention, including the direct costs of the intervention as well as the costs of derivative interventions in the health care and social system. The indirect costs of productivity loss will also be estimated. The time horizon of the evaluation will be 24 months.

The direct costs of both interventions will be estimated based on information collected in the electronic patient records about the actual time spent on delivering the intervention.

Other individual health care costs for each individual will be identified using national register data, which are known to be of high quality and characterized by a high degree of completeness and validity. Individual Civil Registration number (CPR) will be linked to information in the national registers covering all primary and other secondary health care utilization. The indirect costs will be based on the participants’ labor market situation from the Danish DREAM database that holds the unique data of individual sickness absence, compensation benefits, and other social transfer payments.

#### Assessment of safety

All serious adverse events will be recorded and analysed using Barnard’s test for significant differences between intervention groups. We will report the proportion of participants with one or more serious adverse events in both groups. We will use the International Conference on Harmonisation of technical requirements for registration of pharmaceuticals for human use—Good Clinical Practice (ICH-GCP) definition of a serious adverse event, which is any untoward medical occurrence that resulted in death, was life-threatening, required hospitalization or prolonging of existing hospitalization, and resulted in persistent or significant disability or jeopardized the participant [83]. Two investigators will independently go through the participants’ medical journals and assess possible serious adverse events at the 12- and 24-month time point of assessment according to the ICH-GCP definition.

### Interim analyses {21b}

When 50% of the 6 months follow-up data have been collected, interim analyses will be undertaken by an external data safety monitoring committee. The committee will decide whether to stop or continue the trial according to the early stopping criteria by Jakobsen et al. [84], with particular focus on findings of serious adverse effects of treatment. This is in accordance with good clinical practice guidelines of the International Council for Harmonisation of Technical Requirements for Pharmaceuticals for Human Use [83].

### Methods for additional analyses (e.g. subgroup analyses) {20b}

Subgroup analyses to detect symptom deterioration will be performed, even in the case of no statistically significant difference between treatments [85]. Subgroup analyses will be performed for depression symptoms at baseline (taking into account regression to the mean in the interpretation of results), center, sex (male, female), childhood adversity, and psychiatric comorbidities as measured in the M.I.N.I. interview (present; not present). Subgroup effects will be investigated by adding the respective treatment-by-covariate interaction terms to the primary analysis model and by illustrating the subgroup-specific therapy effects in forest plots. More details of the analytical methods will be specified in the forthcoming SAP.

### Methods in analysis to handle protocol non-adherence and any statistical methods to handle missing data {20c}

The primary analysis will be based on the intention-to-treat principle, i.e. data for all randomized participants will be analysed, with conservative imputation of missing outcomes [86]. In a sensitivity analysis, multiple imputation procedures will be used to include dropout status and secondary outcomes in the imputation model. Details about missing data handling will be reported in the forthcoming detailed statistical analysis plan.

### Plans to give access to the full protocol, participant-level data, and statistical code {31c}

Access to full protocol, dataset, and statistical code will be granted upon reasonable request, pending approval from local data protection authorities.

## Oversight and monitoring

### Composition of the coordinating centre and trial steering committee {5d}

The Sponsor-Investigator (a clinical psychologist, PhD, research group leader) and Principal Investigator (PhD-student, clinical psychologist) undertake the day-to-day trial management and as such have regular, weekly meetings, and ad hoc contact as necessary. They are in close contact with the leaders of the four participating clinics, with whom bi-monthly status meetings are held. Key tasks are design of the trial, writing up of the protocol and information for trial participants, applications for legal and ethical approval, setting up education and supervision for clinicians, recruitment and randomization of applicable participants, day-to-day contact with clinics and clinicians, and write up and submittal of outcome articles. The research management team is supported by three PhD co-supervisors with experience in different areas related to the trial who function as consultants throughout the project, and a small number of research and student assistants (Psychology Master’s students) who aid in performing recruitment, inclusion and follow-up measurements.

The statistical analyses of the project data will be made by a statistician (PhD, biostatistician) in collaboration with the PhD-student.

The members of the project steering group will aid in decision making, supervise, and support the trial execution and dissemination of results throughout. The steering group will meet at key stages during the course of the project, at least once a year and allowing for additional meetings as necessary, ensuring that the protocol is followed and taking major strategic decisions. All four trial sites have a representative in the steering committee. The statistician is also a member of the committee.

### Composition of the data monitoring committee, its role and reporting structure {21a}

The data monitoring committee consists of two researchers with experience in the field of psychotherapy and randomized clinical trials. The members are independent from both the sponsor, the conduct of the trial, and any other competing interests. See item 21b for further details.

### Adverse event reporting and harms {22}

ST is not expected to have a higher frequency of or more severe side effects that have been found for other types of psychotherapy. However, we will report all adverse events for trial participants as described in item 20a.

Adverse events are systematically recorded in the Danish health care system records, both those of a somatic and a psychiatric nature. Such events will therefore be recorded in the trial database throughout the trial, regardless of whether the effects can be regarded as caused by the intervention. However, in the case an event happens before the initiation of treatment, this will be recorded as unrelated to the intervention.

All serious, adverse events, or harms possibly related to the trial, e.g. suicidal attempts, admittance to a psychiatric ward, or substantial self-harm, will be reported to the Region Research Ethics Committee immediately, and an annual safety report will be submitted to the Committee, listing of any potential trial-related adverse events or harms.

Also, the Negative Effects Questionnaire, as a part of the secondary outcomes, will address adverse effects of treatment more broadly to qualify which effects are potential harms of treatment and which are unrelated to treatment.

Further, as recommended by the European Medicines Agency [85], subgroup analyses to detect symptom deterioration will be performed to ensure that subgroups with a differential *negative* effect of treatment are detected to provide contraindications towards a particular treatment for certain patients [87].

### Frequency and plans for auditing trial conduct {23}

Additional auditing, independent of the trial investigators, is not planned for this trial.

### Plans for communicating important protocol amendments to relevant parties (e.g. trial participants, ethical committees) {25}

Decisions about protocol amendments are made by the project management group, and, in the case of substantial changes, e.g. in sample size calculations, they will need to be approved by the project steering committee.

All amendments will be submitted to the Region Research Ethics Committee for prior approval before implementation. Revisions directly affecting enrolled trial participants, i.e. changes in treatment, will be communicated to participants by either phone call or through the trial therapists.

Amendments after the registration at Clinicaltrials.gov will be entered directly as revisions to the initial registration.

### Dissemination plans {31a}

#### Planned publications

A number of peer-reviewed scientific articles are planned for the trial, describing the statistical analyses and trial results of the primary, secondary, and exploratory outcomes, as well as the health economic analysis. Results will be submitted for publication regardless of the magnitude or direction of effect. There are no restrictions in the rights for the project group to publish trial results.

Publications and short summaries thereof will be e-mailed to the participating clinicians, as well as participants that express a wish thereof on the trial consent form.

#### Dissemination and planning for clinical utility

##### Public secondary Mental Health Services

The results of the trial will be presented at any interested clinics treating patients with MDD in the Capital and Southern Regions of Denmark.

Further, the Principal Investigator and several of the heads of clinics and clinicians in the trial are currently taking active part in a task force on the treatment of MDD in the Capital Region of Denmark. In this task force, current evidence is applied directly in specific recommendations for clinical education and practice, as well as for future research. It is the hope that the expertise and knowledge acquired in this trial will form the basis of a future, similar task force for DTD.

##### Scientific and clinical community

To reach an international audience, results from the trial will be published in scientific, peer-reviewed journals. Further, participation in a number of international conferences is planned, in which the trial protocol and design, as well as results of the trial and related articles, will be presented.

The Danish scientific community will be informed of the trial and its results through the participation and presentation at several national clinical and scientific conferences. As many of these conferences are open to clinical practitioners, the results of the trial will hereby also reach a clinical audience.

##### Patients and next-of-kin

It is an important aim for the trialists to ensure that the interests of the people whom this trial is aiming to help are heard and taken to consideration. Information and knowledge about the trial, its proceedings and results will be disseminated to patient organizations via a press release at the beginning of the inclusion period as well as an offer of meetings or presentation of results after the trial results have been submitted for publication. Relevant organizations would be: The Depression Association (Depressionsforeningen), The Danish Association for Mental Health (SIND – Landsforeningen for psykisk sundhed), and The Danish Mental Health Fund (Psykiatrifonden).

Further, patients can potentially inform on what are the most important results and implications from the trial from their perspective. Therefore, patient representatives will be approached as consultants for development of the dissemination strategy and focus.

##### Public channels

Two press releases will be written, one at the beginning of the trial period to increase interest in and inform about the prospects of the trial, and one at the end with content about its results and possible implications for patients and treatment.

A more elaborate summary report will also be released and pushed forward in an attempt to be invited to talk about the project and increase visibility for the group of DTD patients in major press channels, such as the Danish Broadcasting Company (DR).

Finally, a web page (www.depre-st.dk) will be launched with information about the trial aims and results for the general public as well as professionals and patients.

## Discussion

The results of this trial can potentially aid in several important aspects of knowledge about psychotherapeutic treatment. Firstly, it will be a much-needed addition to the knowledge about treatment options for patients with DTD. Secondly, it will contribute to expand the evidence base for ST as an emerging, promising treatment for non-personality disorders.

It is the authors’ hope that positive results about the effectiveness of ST for DTD can also lead to a wider implementation of ST as a treatment option in the Danish psychiatric sector. However, given that the direct costs of the investigated ST-treatment are higher than for TAU, this will depend firstly on the results of the health economic analysis, and secondly on the willingness of policy makers to invest in implementing treatment differentiation in MDD patients to achieve better treatment outcomes long term.

The trial has several strengths, given its elaborated, state-of-the-art methodology for randomized clinical trials. This includes blinding of all possible parties, an observer-rated primary outcome, and an investigation of treatment adherence and fidelity for the ST-arm. Further, the external validity is high, given the inclusive participant eligibility criteria and the naturalistic setting of the trial, including the fact that the treatment is provided by non-specialized mental health workers with only limited training.

However, the trial also has limitations. First, we expect large proportions of missing data as is often the case in trials with outcomes reliant on the continuous collaboration of participants, which should warrant cautious interpretations of the results. Second, we pre-defined a minimal important difference of 2 points on the HAM-D-6 scale. However, this minimal important difference is speculative and could be smaller or larger than 2 points. This has to be considered when interpreting the results. Third, the differential length of ST and TAU means that the trial results cannot be interpreted with certainty as to whether any superior effect is due to ST or due to the more extensive treatment alone. However, for pragmatic and monetary reasons, it was not possible for the participating sites to extend the TAU to the same length and format as ST.

Finally, the limited control with TAU, given the pragmatic nature of the trial, also creates heterogeneity in the treatment provided in the TAU-arm, both within and across centres.

## Trial status

This protocol is written based on the registration in ClinicalTrials.gov on 15th April 2023 and the 6th edition of the trial protocol, dated 30th October 2023.

Recruitment of participants began on the 17th April 2023 and is planned to be completed in January 2026.

## Supplementary Information

**Additional file 1.** Table of reviews of psychotherapeutic treatment for difficult-to-treat depression. Table includes characteristics of reviews and most important conclusions.

**Additional file 2.** Approval of study from the Research Ethics Committee of Southern Denmark. Approval of original and revised study protocol and study documents from the Research Ethics Committee of Southern Denmark – in Danish and English translation.

**Additional file 3.** Participant consent form. Participant information and consent form, as approved by the Research Ethics Committee of Southern Denmark – in Danish and English translation.

**Additional file 4.** Details about randomization.

**Additional file 5.** Outcome forms*. *Outcomes (with the exception of forms with copyright, i.e. Childhood Trauma Questionnaire, Symptom Checklist-5, Young Schema Questionnaire, Schema Mode Inventory).

**Additional file 6.** Letters of funding.Letters of funding in DKK from Trygfonden and the Region of Southern Denmark PhD-fund – Danish and English translation.

## Data Availability

Data will be shared with the project statistician and the Data safety monitoring committee, with blinding of any identifying participant information. The Site Investigators can also have access to the collected data upon request.

## Abbreviations

BDI-II: Beck Depression Inventory-II
CBT: Cognitive Behaviour Therapy
CORE-OM: The CORE Outcome Measure
CTQ-SF: The Childhood Trauma Questionnaire Short Form
DAR: Dimensions of Anger Reactions – Revised
DCES-P: Depression Change Expectancy Scale-Pessimistic
DTD: Difficult-to-Treat Depression
ECT: Electroconvulsive therapy
EMS: Early maladaptive schemas
EQ-5D-5L: European Quality of Life 5 Dimensions 5 Level Version
HADS: Hospital Anxiety Depression Scale
HAMD-6: 6 item Hamilton Rating Scale for Depression
ICD-10: International Classification of Diseases 10th Revision
ISST: Internation Society for Schema Therapy
KAG: Clinical academic group
MAP-9: Metacognitive Anger Processing scale Short Version
MDD: Major depressive disorder
MSM: Maudsley Staging Model
NEQ: Negative Effects Questionnaire
PSYCHLOPS: Psychological Outcome Profiles
PTQ: Perseverative Thinking Questionnaire
RNT: Repetitive Negative Thinking
SAP: Statistical analysis plan
SCL-10: Symptom Checklist-10
SMI: Schema Mode Inventory 1.1
SPIRIT: Standard Protocol Items: Recommendations for International Trials
ST: Schema therapy
TAU: Treatment as usual
VAS: Visual analogue scale
WHO: World Health Organization
WHO-5: WHO-5 Well-Being Index
WSAS: Work and Social Adjustment Scale
YSQ-S3: Young Schema Questionnaire 3 Short Form

## References

[1] World Health O. The ICD-10 classification of mental and behavioural disorders: Diagnostic criteria for research. Genève, Switzerland: World Health Organization; 1993. p. 1993.

[2] Van Bronswijk S, Moopen N, Beijers L, Ruhe HG, Peeters F. Effectiveness of psychotherapy for treatment-resistant depression: a meta-analysis and meta-regression. Psychol Med. 2019;49(3):366–79. 30139408 10.1017/S003329171800199X

[3] Wittchen HU, Jacobi F, Rehm J, Gustavsson A, Svensson M, Jönsson B, et al. The size and burden of mental disorders and other disorders of the brain in Europe 2010. Eur Neuropsychopharmacol. 2011;21(9):655–79. 21896369 10.1016/j.euroneuro.2011.07.018

[4] Mairey I, Rosenkilde S, Klitgaard M, Thygesen L. Sygdomsbyrden i Danmark – sygdomme. Udgivet af Sundhedsstyrelsen. København: Statens Institut for Folkesundhed, Syddansk Universitet. 2022.

[5] Sundhedsstyrrelsen. National klinisk retningsline for non-farmakologisk behandling af unipolar depression. 2016.

[6] McAllister-Williams RH, Arango C, Blier P, Demyttenaere K, Falkai P, Gorwood P, et al. The identification, assessment and management of difficult-to-treat depression: An international consensus statement. J Affect Disord. 2020;267:264–82. 32217227 10.1016/j.jad.2020.02.023

[7] Demyttenaere K, Van Duppen Z. The Impact of (the Concept of) Treatment-Resistant Depression: An Opinion Review. Int J Neuropsychopharmacol. 2019;22(2):85–92. 29961822 10.1093/ijnp/pyy052 PMC6368367

[8] Moeller SB, Gbyl K, Hjorthøj C, Andreasen M, Austin SF, Buchholtz PE, et al. Treatment of difficult-to-treat depression – clinical guideline for selected interventions. Nord J Psychiatry. 2022;76(3):177–88. 34455900 10.1080/08039488.2021.1952303

[9] Trevino K, McClintock SM, McDonald Fischer N, Vora A, Husain MM. Defining treatment-resistant depression: a comprehensive review of the literature. Ann Clin Psychiatry. 2014;26(3):222–32. 25166485

[10] Gibson TB, Jing Y, Smith Carls G, Kim E, Bagalman JE, Burton WN, et al. Cost burden of treatment resistance in patients with depression. Am J Manag Care. 2010;16(5):370–7. 20469957

[11] Pfeiffer PN, Kim HM, Ganoczy D, Zivin K, Valenstein M. Treatment-Resistant Depression and Risk of Suicide. Suicide and Life-Threatening Behavior. 2013;43(4):356–65. 23510005 10.1111/sltb.12022

[12] Carney RM, Freedland KE. Treatment-resistant depression and mortality after acute coronary syndrome. Am J Psychiatry. 2009;166(4):410–7. 19289455 10.1176/appi.ajp.2008.08081239 PMC3465939

[13] Jaffe DH, Rive B, Denee TR. The humanistic and economic burden of treatment-resistant depression in Europe: a cross-sectional study. BMC Psychiatry. 2019;19(1):247. 31391065 10.1186/s12888-019-2222-4 PMC6686569

[14] Barnhofer T, Brennan K, Crane C, Duggan D, Williams JM. A comparison of vulnerability factors in patients with persistent and remitting lifetime symptom course of depression. J Affect Disord. 2014;152–154:155–61. 24183488 10.1016/j.jad.2013.09.001 PMC3878770

[15] Negele A, Kaufhold J, Kallenbach L, Leuzinger-Bohleber M. Childhood trauma and its relation to chronic depression in adulthood. Depress Res Treat. 2015;2015: 650804. 26693349 10.1155/2015/650804 PMC4677006

[16] Riso LP, Miyatake RK, Thase ME. The search for determinants of chronic depression: a review of six factors. J Affect Disord. 2002;70(2):103–15. 12117622 10.1016/s0165-0327(01)00376-7

[17] Nelson J, Klumparendt A, Doebler P, Ehring T. Childhood maltreatment and characteristics of adult depression: meta-analysis. Br J Psychiatry. 2017;210(2):96–104. 27908895 10.1192/bjp.bp.115.180752

[18] Köhler S, Chrysanthou S, Guhn A, Sterzer P. Differences between chronic and nonchronic depression: Systematic review and implications for treatment. Depress Anxiety. 2019;36(1):18–30. 30300454 10.1002/da.22835

[19] Hölzel L, Härter M, Reese C, Kriston L. Risk factors for chronic depression — A systematic review. J Affect Disord. 2011;129(1):1–13. 20488546 10.1016/j.jad.2010.03.025

[20] American Psychiatric Association. Diagnostic and Statistical Manual of Mental Disorders, 4th Edition, Text Revision (DSM-IV-TR). Washington: American Psychiatric Association; 2000.

[21] Jobst A, Brakemeier EL, Buchheim A, Caspar F, Cuijpers P, Ebmeier KP, et al. European Psychiatric Association Guidance on psychotherapy in chronic depression across Europe. Eur Psychiatry. 2016;33:18–36. 26854984 10.1016/j.eurpsy.2015.12.003

[22] Peeters N, van Passel B, Krans J. The effectiveness of schema therapy for patients with anxiety disorders, OCD, or PTSD: A systematic review and research agenda. Br J Clin Psychol. 2022;61(3):579–97. 34296767 10.1111/bjc.12324 PMC9544733

[23] Joshua PR, Lewis V, Kelty SF, Boer DP. Is schema therapy effective for adults with eating disorders? A systematic review into the evidence. Cogn Behav Ther. 2023;52(3):213–31. 36633136 10.1080/16506073.2022.2158926

[24] Setkowski K, Palantza C, van Ballegooijen W, Gilissen R, Oud M, Cristea IA, et al. Which psychotherapy is most effective and acceptable in the treatment of adults with a (sub)clinical borderline personality disorder? A systematic review and network meta-analysis. Psychol Med. 2023;53(8):3261–80. 37203447 10.1017/S0033291723000685 PMC10277776

[25] Carter JD, McIntosh VV, Jordan J, Porter RJ, Frampton CM, Joyce PR. Psychotherapy for depression: a randomized clinical trial comparing schema therapy and cognitive behavior therapy. J Affect Disord. 2013;151(2):500–5. 23870427 10.1016/j.jad.2013.06.034

[26] Kopf-Beck J, Müller CL, Tamm J, Fietz J, Rek N, Just L, et al. Effectiveness of Schema Therapy versus Cognitive Behavioral Therapy versus Supportive Therapy for Depression in Inpatient and Day Clinic Settings: A Randomized Clinical Trial. Psychother Psychosom. 2024;93(1):24-35. 10.1159/000535492 PMC10880804 38176391

[27] Malogiannis IA, Arntz A, Spyropoulou A, Tsartsara E, Aggeli A, Karveli S, et al. Schema therapy for patients with chronic depression: A single case series study. J Behav Ther Exp Psychiatry. 2014;45(3):319–29. 24650608 10.1016/j.jbtep.2014.02.003

[28] Renner F, Arntz A, Peeters FPML, Lobbestael J, Huibers MJH. Schema therapy for chronic depression: Results of a multiple single case series. J Behav Ther Exp Psychiatry. 2016;51:66–73. 26780673 10.1016/j.jbtep.2015.12.001

[29] Davies P, Ijaz S, Williams CJ, Kessler D, Lewis G, Wiles N. Pharmacological interventions for treatment-resistant depression in adults. Cochrane Database Syst Rev. 2019;12(12):CD010557. 10.1002/14651858.CD010557.pub2 PMC6916711 31846068

[30] Scott F, Hampsey E, Gnanapragasam S, Carter B, Marwood L, Taylor RW, et al. Systematic review and meta-analysis of augmentation and combination treatments for early-stage treatment-resistant depression. J Psychopharmacol. 2023;37(3):268–78. 35861202 10.1177/02698811221104058 PMC10076341

[31] Cuijpers P, van Straten A, Schuurmans J, van Oppen P, Hollon SD, Andersson G. Psychotherapy for chronic major depression and dysthymia: A meta-analysis. Clin Psychol Rev. 2010;30(1):51–62. 19781837 10.1016/j.cpr.2009.09.003

[32] Negt P, Brakemeier EL, Michalak J, Winter L, Bleich S, Kahl KG. The treatment of chronic depression with cognitive behavioral analysis system of psychotherapy: a systematic review and meta-analysis of randomized-controlled clinical trials. Brain Behav. 2016;6(8): e00486. 27247856 10.1002/brb3.486 PMC4864084

[33] Zakhour S, Nardi AE, Levitan M, Appolinario JC. Cognitive-behavioral therapy for treatment-resistant depression in adults and adolescents: a systematic review. Trends Psychiatry Psychother. 2020;42(1):92–101. 32130308 10.1590/2237-6089-2019-0033

[34] Li JM, Zhang Y, Su WJ, Liu LL, Gong H, Peng W, et al. Cognitive behavioral therapy for treatment-resistant depression: A systematic review and meta-analysis. Psychiatry Res. 2018;268:243–50. 30071387 10.1016/j.psychres.2018.07.020

[35] Ijaz S, Davies P, Williams CJ, Kessler D, Lewis G, Wiles N. Psychological therapies for treatment-resistant depression in adults. Cochrane Database Syst Rev. 2018;5(5):Cd010558. 10.1002/14651858.CD010558.pub2 PMC6494651 29761488

[36] Guyatt GH, Oxman AD, Vist GE, Kunz R, Falck-Ytter Y, Alonso-Coello P, et al. GRADE: an emerging consensus on rating quality of evidence and strength of recommendations. BMJ. 2008;336(7650):924–6. 18436948 10.1136/bmj.39489.470347.AD PMC2335261

[37] Brok J, Thorlund K, Gluud C, Wetterslev J. Trial sequential analysis reveals insufficient information size and potentially false positive results in many meta-analyses. J Clin Epidemiol. 2008;61(8):763–9. 18411040 10.1016/j.jclinepi.2007.10.007

[38] Imberger G, Thorlund K, Gluud C, Wetterslev J. False-positive findings in Cochrane meta-analyses with and without application of trial sequential analysis: an empirical review. BMJ Open. 2016;6(8): e011890. 27519923 10.1136/bmjopen-2016-011890 PMC4985805

[39] Wetterslev J, Jakobsen JC, Gluud C. Trial Sequential Analysis in systematic reviews with meta-analysis. BMC Med Res Methodol. 2017;17(1):39. 28264661 10.1186/s12874-017-0315-7 PMC5397700

[40] Wetterslev J, Thorlund K, Brok J, Gluud C. Trial sequential analysis may establish when firm evidence is reached in cumulative meta-analysis. J Clin Epidemiol. 2008;61(1):64–75. 18083463 10.1016/j.jclinepi.2007.03.013

[41] Cuijpers P, Miguel C, Harrer M, Plessen CY, Ciharova M, Ebert D, et al. Cognitive behavior therapy vs. control conditions, other psychotherapies, pharmacotherapies and combined treatment for depression: a comprehensive meta-analysis including 409 trials with 52,702 patients. World Psychiatry. 2023;22(1):105–15. 10.1002/wps.21069 PMC9840507 36640411

[42] Cuijpers P, Miguel C, Harrer M, Plessen CY, Ciharova M, Papola D, et al. Psychological treatment of depression: A systematic overview of a “Meta-Analytic Research Domain.” J Affect Disord. 2023;335:141–51. 37178828 10.1016/j.jad.2023.05.011

[43] Arntz A, Jacob G. Schema therapy in practice: An introductory guide to the schema mode approach. Hoboken, NJ, US: Wiley Blackwell; 2013. ix, 265-ix, p.

[44] Renner F, Arntz A, Leeuw I, Huibers M. Treatment for Chronic Depression Using Schema Therapy. Clin Psychol Sci Pract. 2013;20(2):166–80.

[45] Bishop A, Younan R, Low J, Pilkington PD. Early maladaptive schemas and depression in adulthood: A systematic review and meta-analysis. Clin Psychol Psychother. 2022;29(1):111–30. 34131990 10.1002/cpp.2630

[46] Sheehan DV, Lecrubier Y, Sheehan KH, Amorim P, Janavs J, Weiller E, et al. The Mini-International Neuropsychiatric Interview (M.I.N.I.): the development and validation of a structured diagnostic psychiatric interview for DSM-IV and ICD-10. J Clin Psychiatry. 1998;59 Suppl 20:22–33;quiz 4–57. 9881538

[47] Danske Regioner. Pakkeforløb for periodisk depression [Available from: https://www.regioner.dk/media/11312/pakkeforloeb-for-periodisk-depression.pdf Accessed 1st Dec 2023.

[48] Danish Patient Compensation. [Available from: https://eng.patienterstatningen.dk/ Accessed 4 Jan 2024.

[49] Licht RW, Qvitzau S, Allerup P, Bech P. Validation of the Bech-Rafaelsen Melancholia Scale and the Hamilton Depression Scale in patients with major depression; is the total score a valid measure of illness severity? Acta Psychiatr Scand. 2005;111(2):144–9. 15667434 10.1111/j.1600-0447.2004.00440.x

[50] Peasgood T, Brazier J, Papaioannou D. A systematic review of the validity and responsiveness of EQ-5D and SF-6D for depression and anxiety. HEDS Discussion paper 12/15. 2012.

[51] Sørensen J, Davidsen M, Gudex C, Pedersen KM, Brønnum-Hansen H. Danish EQ-5D population norms. Scand J Public Health. 2009;37(5):467–74. 19535407 10.1177/1403494809105286

[52] Mundt JC, Marks IM, Shear MK, Greist JH. The Work and Social Adjustment Scale: a simple measure of impairment in functioning. Br J Psychiatry. 2002;180:461–4. 11983645 10.1192/bjp.180.5.461

[53] Topp CW, Østergaard SD, Søndergaard S, Bech P. The WHO-5 Well-Being Index: a systematic review of the literature. Psychother Psychosom. 2015;84(3):167–76. 25831962 10.1159/000376585

[54] Rozental A, Castonguay L, Dimidjian S, Lambert M, Shafran R, Andersson G, et al. Negative effects in psychotherapy: commentary and recommendations for future research and clinical practice. BJPsych Open. 2018;4(4):307–12. 30083384 10.1192/bjo.2018.42 PMC6066991

[55] Rozental A, Kottorp A, Forsström D, Månsson K, Boettcher J, Andersson G, et al. The Negative Effects Questionnaire: psychometric properties of an instrument for assessing negative effects in psychological treatments. Behav Cogn Psychother. 2019;47(5):559–72. 30871650 10.1017/S1352465819000018

[56] Williams J, Leamy M, Bird V, Le Boutillier C, Norton S, Pesola F, et al. Development and evaluation of the INSPIRE measure of staff support for personal recovery. Soc Psychiatry Psychiatr Epidemiol. 2015;50(5):777–86. 25409867 10.1007/s00127-014-0983-0

[57] Moeller SB, Gondan M, Austin SF, Slade M, Simonsen S. National norms of mental health for Denmark. Nord J Psychiatry. 2023;77(6):617–23. 37129238 10.1080/08039488.2023.2202637

[58] Williams J, Leamy M, Bird V, Harding C, Larsen J, Le Boutillier C, et al. Measures of the recovery orientation of mental health services: systematic review. Soc Psychiatry Psychiatr Epidemiol. 2012;47(11):1827–35. 22322983 10.1007/s00127-012-0484-y

[59] Ashworth M, Shepherd M, Christey J, Matthews V, Wright K, Parmentier H, et al. A client-generated psychometric instrument: The development of “PSYCHLOPS.” Couns Psychother Res. 2004;4(2):27–31.

[60] Ashworth M, Evans C, Clement S. Measuring psychological outcomes after cognitive behaviour therapy in primary care: A comparison between a new patient-generated measure “PSYCHLOPS” (Psychological Outcome Profiles) and “HADS” (Hospital Anxiety and Depression Scale). J Ment Health. 2009;18(2):169–77.

[61] Ashworth M, Robinson SI, Godfrey EL, Shepherd M, Evans CH, Seed PT, et al., editors. Measuring mental health outcomes in primary care: The psychometric properties of a new patient-generated outcome measure, 'PSYCHLOPS' ('psychological outcome profiles')2005.

[62] Kannis-Dymand L, Salguero JM, Ramos-Cejudo J, Novaco RW. Dimensions of Anger Reactions-Revised (DAR-R): Validation of a brief anger measure in Australia and Spain. J Clin Psychol. 2019;75(7):1233–48. 30758849 10.1002/jclp.22757

[63] Moeller SB, Juul S, Arendt ITP. The Short Version of the Metacognitive Anger Processing Scale (MAP-SV) - initial psychometric testing. Behav Cogn Psychother. 2022;50(1):117–21. 34078508 10.1017/S1352465821000199

[64] Ehring T, Zetsche U, Weidacker K, Wahl K, Schönfeld S, Ehlers A. The Perseverative Thinking Questionnaire (PTQ): validation of a content-independent measure of repetitive negative thinking. J Behav Ther Exp Psychiatry. 2011;42(2):225–32. 21315886 10.1016/j.jbtep.2010.12.003 PMC3042595

[65] Moeller SB, Larsen PV, Arendt I-MTP, Ehring T, Reinholt N, Hvenegaard M, et al. Validation of the Danish Version of Perseverative Thinking Questionnaire (PTQ) – Introducing the PTQ Short Version. Psychol Test Adapt Dev. 2023;4(1):310–8.

[66] Bech P, Austin SF, Lau ME. Patient reported outcome measures (PROMs): examination of the psychometric properties of two measures for burden of symptoms and quality of life in patients with depression or anxiety. Nord J Psychiatry. 2018;72(4):251–8. 29546787 10.1080/08039488.2018.1451918

[67] Østergaard SD, Papakostas GI, Fava M. Depression: Response and Remission. In: Stolerman IP, Price LH, editors. Encyclopedia of Psychopharmacology. Berlin, Heidelberg: Springer Berlin Heidelberg; 2015. p. 505-9.

[68] Lobbestael J, van Vreeswijk M, Spinhoven P, Schouten E, Arntz A. Reliability and validity of the short Schema Mode Inventory (SMI). Behav Cogn Psychother. 2010;38(4):437–58. 20487590 10.1017/S1352465810000226

[69] Reiss N, Krampen D, Christoffersen P, Bach B. Reliability and validity of the Danish version of the Schema Mode Inventory (SMI). Psychol Assess. 2016;28(3):e19–26. 26375430 10.1037/pas0000154

[70] Bach B, Simonsen E, Christoffersen P, Kriston L. The Young Schema Questionnaire 3 Short Form (YSQ-S3): Psychometric properties and association with personality disorders in a Danish mixed sample. Eur J Psychol Assess. 2017;33(2):134–43.

[71] Fekadu A, Wooderson S, Donaldson C, Markopoulou K, Masterson B, Poon L, et al. A multidimensional tool to quantify treatment resistance in depression: the Maudsley staging method. J Clin Psychiatry. 2009;70(2):177–84. 19192471 10.4088/jcp.08m04309

[72] Bernstein DP, Stein JA, Newcomb MD, Walker E, Pogge D, Ahluvalia T, et al. Development and validation of a brief screening version of the Childhood Trauma Questionnaire. Child Abuse Negl. 2003;27(2):169–90. 12615092 10.1016/s0145-2134(02)00541-0

[73] Kongerslev MT, Bach B, Rossi G, Trauelsen AM, Ladegaard N, Løkkegaard SS, et al. Psychometric validation of the Childhood Trauma Questionnaire-Short Form (CTQ-SF) in a Danish clinical sample. Child Abuse Negl. 2019;94: 104026. 31154112 10.1016/j.chiabu.2019.104026

[74] Dozois DJ, Westra HA. Development of the Anxiety Change Expectancy Scale (ACES) and validation in college, community, and clinical samples. Behav Res Ther. 2005;43(12):1655–72. 15922290 10.1016/j.brat.2004.12.001

[75] Eddington KM, Dozois DJ, Backs-Dermott BJ. Evaluation of the internal consistency, factor structure, and validity of the Depression Change Expectancy Scale. Assessment. 2014;21(5):607–17. 24379447 10.1177/1073191113517929

[76] Østergaard SD, Bech P, Miskowiak KW. Fewer study participants needed to demonstrate superior antidepressant efficacy when using the Hamilton melancholia subscale (HAM-D_6_) as outcome measure. J Affect Disord. 2016;190:842–5. 25487682 10.1016/j.jad.2014.10.047

[77] Timmerby N, Andersen JH, Søndergaard S, Østergaard SD, Bech P. A Systematic Review of the Clinimetric Properties of the 6-Item Version of the Hamilton Depression Rating Scale (HAM-D6). Psychother Psychosom. 2017;86(3):141–9. 28490031 10.1159/000457131

[78] Rush AJ, South C, Jain S, Agha R, Zhang M, Shrestha S, et al. Clinically significant changes in the 17- and 6-item hamilton rating scales for depression: A STAR*D Report. Neuropsychiatr Dis Treat. 2021;17:2333–45. 34295161 10.2147/NDT.S305331 PMC8290193

[79] Faul F, Erdfelder E, Lang A-G, Buchner A. G*Power 3: A flexible statistical power analysis program for the social, behavioral, and biomedical sciences. Behav Res Methods. 2007;39(2):175–91. 17695343 10.3758/bf03193146

[80] Sealed Envelope Ltd. Create a blocked randomization list. 2022 [Available from: https://sealedenvelope.com/simple-randomiser/v1/lists Accessed 10 Feb 2023.

[81] Juul S, Gluud C, Simonsen S, Frandsen FW, Kirsch I, Jakobsen JC. Blinding in randomised clinical trials of psychological interventions: a retrospective study of published trial reports. BMJ Evidence-Based Medicine. 2021;26(3):109. 32998993 10.1136/bmjebm-2020-111407

[82] Committee for Medicinal Products for Human Use. Guideline on adjustment for baseline covariates in clinical trials. London/Amsterdam: European Medicines Agency; 2015.

[83] International council for harmonisation of tehcnical requirements for pharmaceuticals for human use. ICH Harmonised guideline. Good Clinical Practice (GCP) E6(R3). Report No.: Draft version, endorsed on 19 May 2023.

[84] Jakobsen JC, Gluud C, Winkel P, Lange T, Wetterslev J. The thresholds for statistical and clinical significance – a five-step procedure for evaluation of intervention effects in randomised clinical trials. BMC Med Res Methodol. 2014;14(1):34. 24588900 10.1186/1471-2288-14-34 PMC4015863

[85] Committee for Medicinal Products for Human Use. Guideline on the investigation of subgroups in confirmatory clinical trials. London/Amsterdam: Eur Med Agency. 2019.

[86] Wolbers M, Noci A, Delmar P, Gower-Page C, Yiu S, Bartlett JW. Standard and reference-based conditional mean imputation. Pharm Stat. 2022;21(6):1246–57. 35587109 10.1002/pst.2234 PMC9790242

[87] Linden M, Schermuly-Haupt ML. Definition, assessment and rate of psychotherapy side effects. World Psychiatry. 2014;13(3):306–9. 25273304 10.1002/wps.20153 PMC4219072

