# Supplementary material for: Schema therapy versus treatment as usual for outpatients with difficult-to-treat depression: study protocol for a parallel group randomized clinical trial (DEPRE-ST)
Source: Trials. 2024 Apr 16;25:266. doi: 10.1186/s13063-024-08079-9 (PMC11022394; doi:10.1186/s13063-024-08079-9)
Supplement: Supplementary file 1 — Additional file 1. Table of reviews of psychotherapeutic treatment for difficult-to-treat depression. Table includes characteristics of reviews and most important conclusions. [file 13063_2024_8079_MOESM1_ESM.docx]

| **Authors** | **Publication year** | **Only RCTs included** | **Databases searched** | **No. of studies included** | **No. of patients included** | **Pre-published protocol** | **Patient group** | **Psychological interventions (no of trials)** |
| --- | --- | --- | --- | --- | --- | --- | --- | --- |
| Van Bronswijk S, Moopen N, Beijers L, Ruhe HG, Peeters F (2) | 2019 | Yes | PubMed, Embase, PsycInfo | 22 | 3539 | No | TRD (one failed medication attempt), CD if majority of patients in a study also had TRD | CBT (6), CBASP (6), IPT (2), MBCT (4), DBT(1), ST (1), Occupational therapy 1), IRCBT (1), PBCT (1), GBOPT (1), PDP (1) |
| Cuijpers P, van Straten A, Schuur-mans J, van Oppen P, Hollon SD, Andersson G (30) | 2010 | Yes | PubMed, PsycInfo, Embase, Cochrane Central Register of Controlled Trials | 16 | 2116 | No | CD, dysthymia | CBT (7), IPT (6), PST (2), ST (2), SST (2), CIGP (1); CBASP (1), BA (1) |
| Negt P, Brakemeier EL, Michalak J, Winter L, Bleich S, Kahl KG (31) | 2016 | Yes | PsycInfo, PubMed, Scopus, Cochrane Central Register of Controlled Trials | 6 | 1.510 | No | CD, recurrent depression, dysthymia | CBASP (6) |
| Ijaz S, Davies P, Williams CJ, Kessler D, Lewis G, Wiles N (32) | 2018 | Yes | Cochrane Common Mental Disorders Controlled Trials Register, CENTRAL, MEDLINE, Embase, PsycINFO, World Health Organization (WHO) trials portal (ICTRP), ClinicalTrials.gov | 6 | 698 | Yes | TRD (one failed medication attempt) | CBT (3), IPT (1), ISTDP (1), DBT (1) |
| Li JM, Zhang Y, Su WJ, Liu LL, Gong H, Peng W, et al (33) | 2018 | Yes | PubMed, Embase, and Cochrane Library | 6 | 847 | Yes | TRD (one failed medication attempt) | CBT-based therapies: CBT (2), MBCT (2), RFCBT (1), smartphone-based CBT (1) |
| Zakhour S, Nardi AE, Levitan M, Appolinario JC (34) | 2020 | No, also included open studies and case reports | PubMed, SciELO, Psychiatry Online, Scopus, PsycArticles, Science Direct and the Journal of Medical Case Reports database | 8 | 1056 | No | TRD (one failed medication attempt) | CBT (8) |

| **Authors** | **Comparators** | **Effect size** | **Assessment of adverse events** | **GRADE assessment** | **Risk of Bias assessment** | **TSA** | **Conclusion** |
| --- | --- | --- | --- | --- | --- | --- | --- |
| Van Bronswijk S, Moopen N, Beijers L, Ruhe HG, Peeters F- (2) | TAU (including pharmacotherapy) | *g*=0.42 for Add-on psychotherapy to TAU  *g*=-0.13 for psychotherapy vs TAU | No | No | Yes (four COCHRANE criteria) | No | Good effect of psychotherapy added to TAU. Strongest evidence base for CBT, MBCT, and CBASP. Higher effect sizes for group vs individu-al format and for higher baseline depression severity. |
| Cuijpers P, van Straten A, Schuurmans J, van Oppen P, Hollon SD, Andersson G (30) | Pharmacotherapy/TAU, placebo, Waiting list | *d*=0.23 (addition of psychotherapy to pharmacotherapy only):  *d*=−0.31 (psychotherapy only vs pharmacotherapy);  d = 0.45 (psychotherapy only vs combination of psychotherapy and pharmacotherapy | No | No | Yes (three COCHRANE criteria) | No | Smaller effect of psychotherapy for CD/dysthymia than non-CD.  No difference between psychotherapies.  Combination of psychotherapy and pharmaco-therapy most effective.  >18 sessions required for adequate effect. |
| Negt P, Brakemeier EL, Michalak J, Winter L, Bleich S, Kahl KG (31) | Pharmacotherapy, CBT, IPT, PDP, MBCT, ST | *g*=0.34 | No | No | Yes (three self-selected criteria) | No | CBASP is effective in the treatment of CD. Smaller effects than for psychotherapy in non-CD. |
| Ijaz S, Davies P, Williams CJ, Kessler D, Lewis G, Wiles N (32) | TAU (including pharmacotherapy) | SMD = 0.40 | Yes | Yes | Yes | No | Moderate quality of evidence (GRADE) for short term effects (<6 months) of psychotherapy – low quality of evidence for medium to long-term effects (>6 months), both on self-reported but not observer-rated depressive symptoms. |
| Li JM, Zhang Y, Su WJ, Liu LL, Gong H, Peng W, et al (33) | TAU (including pharmacotherapy) | SMD = -0.42 at end of treatment,  SMD = -0.44 (6 months followup),  SMD= -0.29 (1 year followup) | No | No | Yes | No | CBT + pharmaco-therapy is more effective on depressive symptoms than pharmacotherapy alone up to 1 year follow up. |
| Zakhour S, Nardi AE, Levitan M, Appolinario JC (34) | TAU | Narrative review; none calculated | Yes | No | Yes (Randomized Controlled Trial Psycho-therapy  Quality Rating Scale) | No | CBT + pharmacotherapy is effective for depressive symptoms in TRD. |

BA = Behavioral Activation; CBASP = Cognitive Behavioral Analysis System of Psychotherapy; CBT = Cognitive Behavior Therapy; CIGP = group psychotherapy for chronic depression; DBT, Dialectic Behaviour Therapy; GBOP = Group body oriented psychological therapy; IPT = Interpersonal Therapy; ISTDP = Intensive Short Term Psychodynamic Therapy; MBCT, Mindfulness Based Cognitive Therapy; PBCT = Person-Based Cognitive Therapy; PDP = Psycho-dynamic psychotherapy; Problem-Solving Therapy = PST; RFCBT = Rumination-Focused Cognitive Behaviour Therapy; SMD = Standardized Mean Difference; ST = Supportive Therapy; SST = Social Skills Training; TAU = treatment as usual; TRD = treatment resistant depression TSA = Trial sequential analysis

**Reference to reviews in main article:**

2. Van Bronswijk S, Moopen N, Beijers L, Ruhe HG, Peeters F. Effectiveness of psychotherapy for treatment-resistant depression: a meta-analysis and meta-regression. Psychological Medicine. 2019;49(3):366-79.

30. Cuijpers P, van Straten A, Schuurmans J, van Oppen P, Hollon SD, Andersson G. Psychotherapy for chronic major depression and dysthymia: A meta-analysis. Clinical Psychology Review. 2010;30(1):51-62.

31. Negt P, Brakemeier EL, Michalak J, Winter L, Bleich S, Kahl KG. The treatment of chronic depression with cognitive behavioral analysis system of psychotherapy: a systematic review and meta-analysis of randomized-controlled clinical trials. Brain Behav. 2016;6(8):e00486.

32. Ijaz S, Davies P, Williams CJ, Kessler D, Lewis G, Wiles N. Psychological therapies for treatment-resistant depression in adults. Cochrane Database Syst Rev. 2018;5(5):Cd010558.

33. Li JM, Zhang Y, Su WJ, Liu LL, Gong H, Peng W, et al. Cognitive behavioral therapy for treatment-resistant depression: A systematic review and meta-analysis. Psychiatry Res. 2018;268:243-50.

34. Zakhour S, Nardi AE, Levitan M, Appolinario JC. Cognitive-behavioral therapy for treatment-resistant depression in adults and adolescents: a systematic review. Trends Psychiatry Psychother. 2020;42(1):92-101.
