# Supplementary material for: Schema therapy versus treatment as usual for outpatients with difficult-to-treat depression: study protocol for a parallel group randomized clinical trial (DEPRE-ST)
Source: Trials. 2024 Apr 16;25:266. doi: 10.1186/s13063-024-08079-9 (PMC11022394; doi:10.1186/s13063-024-08079-9)
Supplement: Supplementary file 2 — Additional file 2. Approval of study from the Research Ethics Committee of Southern Denmark. Approval of original and revised study protocol and study documents from the Research Ethics Committee of Southern Denmark – in Danish and English translation. [file 13063_2024_8079_MOESM2_ESM.pdf]

The Scientific ethics Committees for Region Southern Denmark

komite@rsyd.dk

Stine Bjerrum Møller
Department for Torture- and Trauma survivors/Institute for Psychology, Region Southern Denmark Psychiatry/University of Southern Denmark

Date: 14 . November 2022 Project ID S-20220033 Acadre no : 22/24944
Betina Simonsen/ Christina Sølvsten Fly

Research project: DEPRE-ST - one randomized, controlled examination of schema therapy for patients with difficult-to-treat depression.

The Scientific ethics Committee for Region Southern Denmark has the 7. September 2022 received one notification of supplement with notification no . 94755

The supplement deals with: Cf. _ review

Decision
The committee able to approve the above Appendix .
That will say the changes that is described or is indicated as one separately submitted listing on the notification form itself .

The approval applies from of the day date. The committee wishes to do aware of to current approval for the project **is valid until 1 May 2026.**

The approval includes following documents :

- Protocol ver4 dated 6 . September 2022 Received 7 . September 2022
- Participant information ver2A video recordings Received 7. September 2022
- Participant information ver2b Pilot Received 7 . September 2022
- Participant information ver3 incl. Video recordings Received 7. September 2022

Remarks
That noted that the committee does not is departmental authority relating to. the regulation on data protection, and to the committee assumes that the changes are according with the European Parliament and Council Regulation no. 2016/679 of 27 . April 2016 about protection of physical persons in connection with Treatment of personal information and on the free exchange of such information and the data protection act.
The case have been processed and is finally approved by of the committee secretariat

On Committee's on behalf of kind regards

Chairman Aia Elise Jønch, Chief Physician, PhD

Copy to: Ida-Marie T. P. Arendt, imarendt@health.sdu.dk

De Videnskabsetiske Komiteer for Region Syddanmark

komite@rsyd.dk

Stine Bjerrum Møller
Afdeling for Tortur- og Traumeoverlevere/Institut for Psykologi, Region Syddanmark
Psykiatri/Syddansk Universitet

Dato: 14. november 2022 Projekt-ID S-20220033
Acadre nr: 22/24944
Betina Simonsen/ Christina Sølvsten Fly

Forskningsprojekt: DEPRE-ST - en randomiseret, kontrolleret undersøgelse af schematerapi for patienter med svært behandlelig depression.

Den Videnskabsetiske Komite for Region Syddanmark har den 7. september 2022 modtaget en anmeldelse af tillæg med anmeldelses nr. 94755

Tillægget omhandler: Jf. anmeldelse

Afgørelse
Komiteen kan godkende ovenstående tillæg.
Det vil sige de ændringer, der er beskrevet eller er angivet som en særskilt indsendt oplistning på selve anmeldelsesblanketten.

Godkendelsen gælder fra dags dato. Komiteen ønsker at gøre opmærksom på, at nuværende godkendelse for projektet **gælder til 1. maj 2026.**

Godkendelsen omfatter følgende dokumenter:

- Protokol ver4 dateret 6. september 2022 modtaget 7. september 2022
- Deltagerinformation ver2A videooptagelser modtaget 7. september 2022
- Deltagerinformation ver2b Pilot modtaget 7. september 2022
- Deltagerinformation ver3 inkl. Videooptagelser modtaget 7. september 2022

Bemærkninger
Det bemærkes, at komiteen ikke er ressortmyndighed vedr. regelsættet om databeskyttelse, og at komiteen forudsætter, at ændringerne er i overensstemmelse med Europa-Parlamentets og Rådets forordning nr.
2016/679 af 27. april 2016 om beskyttelse af fysiske personer i forbindelse med behandling af person- oplysninger og om fri udveksling af sådanne oplysninger og databeskyttelsesloven.
Sagen har været behandlet og er endelig godkendt af komiteens sekretariat

På Komiteens vegne venlig hilsen

Formand Aia Elise Jønch, Overlæge, phd

Kopi til: Ida-Marie T. P. Arendt, imarendt@health.sdu.dk

The Scientific Ethics Committees
for Region Southern Denmark

komite@rsyd.dk

Stine Bjerrum Møller
Region Southern Denmark Psychiatry/University of Southern Denmark Department for Torture and Trauma Survivors/Institute for Psychology
Vestre Engvej 51 Entrance B, 3rd floor
7100 Vejle

Date: 3. August 2022
Project-ID: S-20220033
Acadren no: 22/24944
Pernille Birk Heby /
Christina Sølvsten Fly

Research project: DEPRE-ST a randomized, controlled study of schema therapy for patients with difficult-to-treat depression

The Scientific Ethics Committee 2 for Region Southern Denmark has now made a final decision on the approval of your project. On 5 July 2022, the committee received revised material. The project material now meets the conditions given by the decision on 16 June 2022.

The decision was made in accordance with Act No. 1338 of 1 September 2020 on scientific ethical treatment of health science research projects (Committee Act). The

approval applies to the notified trial sites, the notified trial managers in Denmark as well as for the stated trial period, the Committee expects the trial manager to ensure that the other participants in the project on the Committee's decision.

The approval is valid from 3 August 2022 to 1 May 2026. After the expiry of the approval, notifiable procedures, such as the collection of new data, may not be carried out. (See section: "Changes").

The approval includes the following documents:

•	Experimental protocol	version 3	
•	Participant information	version 2	July 5, 2022
•	Declaration of consent	version 2	July 5, 2022
•	Questionnaires		
•	Oral participant information	version 1	6, maj 2022
•	Information on the processing of personal data	version 1	5, maj 2022
•	Participant folder	Received	17, maj 2022

Implementation of the project in violation of the committee's approval can be punished with a fine or imprisonment, cf. Sections 41 and 42 of the Committee Act.

Remarks

It should be noted that the committee is not the competent authority regarding the regulations on data protection, and that the committee assumes that the project is carried out in accordance with Regulation No. 2016/679 of the European Parliament and of the Council of 27 April 2016 on the protection of natural persons in connection with the processing of personal data and on the free exchange of such information and the Data Protection Act.

The committee's approval includes permission for information from the patient record to be used in the project i according to Section 46 of the Health Act or Section 3, subsection of the Committee Act. 3. This permission includes the information that is listed in the protocol and participant information respectively.

The Scientific Ethics Committees
for Region Southern Denmark

komite@rsyd.dk

Responsibilities of researchers

As a trial manager, you must be aware of the following obligations in relation to the committee system:

Changes

If the person in charge of the experiment makes significant changes during the implementation of the project, the changes must be notified to the committee in the form of additional protocols. Only when the committee's approval of the changes has been received can these be implemented, cf. section 27 of the Committees Act. 1.

The person in charge of the trial must notify supplementary protocols electronically at www.drvk.dk. In the notification, it must initially assigned notification number and password are used.

See guidance on changes to an approved research project:
http://www.nvk.dk/forsker/forskervejledning/vejledning-om-aendringer-i-et-godkendt-projekt

Continuous reporting of side effects and incidents

The person in charge of the trial must immediately report to the committee if suspected serious, unexpected side effects or serious incidents occur during the project, cf. section 30, subsection of the committee act. 1. The report must be accompanied by comments on any consequences for the trial. Only side effects and incidents that occur in Denmark must be reported. The report must be made no later than seven days after the sponsor or the person in charge of the trial has become aware of the incident.

Honest status report

Once a year during the entire trial period, the trial supervisor must send an annual report to the committee. The report must contain a list of all suspected serious (expected and unexpected) side effects and serious incidents that have occurred during the trial period together with a report on the safety of the test subjects, cf. section 30, subsection of the committee act. 2.

The material must be in Danish or English.

The above reports can be made using forms available at
http://www.nvk.dk/emner/bivirkninger/hvornaar-skal-bivirkninger-indberettes The form
with attachments must be emailed to komite@rsyd.dk.

Supervision

The committee supervises that the project is carried out in accordance with the approval, cf. Sections 28 and 29.

Notification of termination

The person in charge of the trial must notify the committee of this no later than 90 days after the date of expiry of the approval, cf. Section 31, subsection 1 of the Committee Act.

If the experimenter interrupts his project earlier than planned, a reason for this must be sent to the committee at the latest 15 days after the decision has been made, cf. Section 31, subsection of the Committee Act. 2.

If the experimenter does not start his project, this and the reason for this must be communicated to the committee.

Notification can be made using a form available at http://
www.nvk.dk/forsker/indberetning-ved-afslutning-af-forsoeg The form
with attachments must be emailed to komite@rsyd.dk.

If the experimenter does not start his project, this and the reason for this must be communicated to the committee.

We must ask those responsible for the experiment to always state project id S-20220033 when sending project materials to the committee. Inquiries regarding your project can be directed to the committee's secretariat at komite@rsyd.dk.

The matter has been dealt with and has finally been approved by the committee's secretariat

Kind regards on behalf of
the Committee

The Scientific Ethics Committees
for Region Southern Denmark

komite@rsyd.dk

Aia Elise Jønch, General practitioner,
PhD Chairman of Committee 2

Copy to:

Ida-Marie T. P. Arendt imarendt@health.sdu.dk

De Videnskabsetiske Komitéer
for Region Syddanmark

komite@rsyd.dk

Stine Bjerrum Møller
Region Syddanmark Psykiatri/Syddansk Universitet
Afdeling for Tortur- og Traumeoverlevere/Institut for Psykologi
Vestre Engvej 51 Indgang B, 3. sal
7100 Vejle

Dato: 3. august 2022
Projekt-ID: **S-20220033**
Acadrenr: 22/24944.
Pernille Birk Heby /
Christina Sølvsten Fly

Forskningsprojekt: DEPRE-ST - en randomiseret, kontrolleret undersøgelse af schematerapi for patienter med svært behandlelig depression

Den Videnskabsetiske Komité 2 for Region Syddanmark har nu truffet endelig afgørelse om **godkendelse af dit projekt**. Komitéen har den 5. juli 2022 modtaget revideret materiale. Projektmaterialet opfylder nu vilkår givet ved afgørelsen den 16. juni 2022.
Afgørelsen er truffet i henhold til lov nr. 1338 af 1. september 2020 om videnskabsetisk behandling af sundhedsvidenskabelige forskningsprojekter (komiteloven).

Godkendelsen gælder for de anmeldte forsøgssteder, de anmeldte forsøgsansvarlige i Danmark samt for den angivne forsøgsperiode. Komitéen forventer, at den forsøgsansvarlige sørger for at underrette de øvrige deltagere i projektet om Komitéens afgørelse.

Godkendelsen gælder fra 3. august 2022 til den 1. maj 2026. Efter godkendelsens udløb må der ikke foretages anmeldelsespligtige procedurer, såsom indsamling af nye data. (Se afsnit: "Ændringer").

Godkendelsen omfatter følgende dokumenter:

•	Forsøgsprotokol	version 3	
•	Deltagerinformation	version 2	5. juli 2022
•	Samtykkeerklæring	version 2	5. juli 2022
•	Spørgeskemaer		
•	Mundtlig deltagerinformation	version 1	6. maj 2022
•	Oplysning om behandling af personoplysninger	version 1	5. maj 2022
•	Deltagerfolder	modtaget	17. maj 2022

Iværksættelse af projektet i strid med komitéens godkendelse kan straffes med bøde eller fængsel, jf. komitélovens §§41 og 42.

Bemærkninger
Det bemærkes, at komiteen ikke er ressortmyndighed vedr. regelsættet om databeskyttelse, og at komiteen forudsætter, at projektet gennemføres i overensstemmelse med Europa-Parlamentets og Rådets forordning nr. 2016/679 af 27. april 2016 om beskyttelse af fysiske personer i forbindelse med behandling af personoplysninger og om fri udveksling af sådanne oplysninger og databeskyttelsesloven.

Komitéens godkendelse omfatter tilladelse til, at der kan anvendes oplysninger fra patientjournalen i projektet i henhold til sundhedslovens §46 eller komitelovens §3, stk. 3. Denne tilladelse omfatter de oplysninger, der er oplistet i henholdsvis protokol og deltagerinformation.

De Videnskabsetiske Komitéer
for Region Syddanmark

komite@rsyd.dk

Forskers pligter
Som forsøgsansvarlig skal du være opmærksom på følgende forpligtelser i forhold til komitésystemet:

Ændringer
Hvis den forsøgsansvarlige foretager væsentlige ændringer under projektets gennemførelse, skal ændringerne anmeldes til komitéen i form af tillægsprotokoller. Først når komitéens godkendelse af ændringerne er modtaget, må disse iværksættes, jf. komitélovens §27, stk. 1.

Forsøgsansvarlig skal anmelde tillægsprotokoller elektronisk på www.drvk.dk. Ved anmeldelsen skal det oprindeligt tildelte anmeldelsesnummer og adgangskode anvendes.

Se vejledning om ændringer af et godkendt forskningsprojekt:
http://www.nvk.dk/forsker/forskervejledning/vejledning-om-aendringer-i-et-godkendt-projekt

Løbende indberetning af bivirkninger og hændelser
Forsøgsansvarlig skal omgående indberette til komitéen, hvis der under projektet optræder formodet alvorlige, uventede bivirkninger eller alvorlige hændelser, jf. komitélovens §30, stk. 1. Indberetningen skal ledsages af kommentarer om eventuelle konsekvenser for forsøget. Kun bivirkninger og hændelser der forekommer i Danmark, skal indberettes. Indberetningen skal ske senest syv dage efter, at sponsor eller den forsøgsansvarlige har fået kendskab til tilfældet.

Årlig statusindberetning
Én gang årligt i hele forsøgsperioden skal forsøgsansvarlig sende en årlig indberetning til komitéen. Indberetningen skal indeholde en liste over alle formodet alvorlige (ventede og uventede) bivirkninger og alvorlige hændelser, som er indtruffet i forsøgsperioden sammen med en rapport om forsøgspersonernes sikkerhed, jf. komitélovens §30, stk. 2.
Materialet skal være på dansk eller engelsk.

Ovenstående indberetninger kan ske ved hjælp af skemaer, der findes på
http://www.nvk.dk/emner/bivirkninger/hvornaar-skal-bivirkninger-indberettes
Skemaet med bilag skal mailes til komite@rsyd.dk.

Tilsyn:
Komiteen fører tilsyn med, at projektet udføres i overensstemmelse med godkendelsen, jf. komitélovens §§28 og 29.

Underretning om afslutning
Den forsøgsansvarlige skal senest 90 dage efter datoen for godkendelsens udløb underrette komitéen herom, jf. komitélovens §31, stk. 1.

Afbryder forsøgsansvarlig sit projekt tidligere end planlagt, skal en begrundelse herfor sendes til komitéen senest 15 dage efter, at beslutningen er truffet, jf. komitélovens §31, stk. 2.

Hvis forsøgsansvarlig ikke påbegynder sit projekt, skal dette samt årsagen hertil meddeles komiteen.

Underretningen kan ske ved hjælp af et skema, der findes på
http://www.nvk.dk/forsker/indberetning-ved-afslutning-af-forsoeg
Skemaet med bilag skal mailes til komite@rsyd.dk.

Hvis forsøgsansvarlig ikke påbegynder sit projekt, skal dette samt årsagen hertil meddeles komiteen.

Vi skal bede forsøgsansvarlige om altid at anføre **projekt id S-20220033**, ved fremsendelse af projektmaterialer til komitéen. Henvendelser vedrørende dit projekt kan rettes til komitéens sekretariat på komite@rsyd.dk.

Sagen har været behandlet og er endelig godkendt af komitéens sekretariat

På Komiteens vegne
venlig hilsen

Region Syddanmark

Regionshuset, Damhaven 12, 7100 Vejle
Tlf.: 76638221
www.regionsyddanmark.dk/komite

De Videnskabsetiske Komitéer
for Region Syddanmark

komite@rsyd.dk

Aia Elise Jønch, Overlæge, phd
Formand for Komite 2

Kopi til:

Ida-Marie T. P. Arendt imarendt@health.sdu.dk
