# Supplementary material for: Schema therapy versus treatment as usual for outpatients with difficult-to-treat depression: study protocol for a parallel group randomized clinical trial (DEPRE-ST)
Source: Trials. 2024 Apr 16;25:266. doi: 10.1186/s13063-024-08079-9 (PMC11022394; doi:10.1186/s13063-024-08079-9)
Supplement: Supplementary file 3 — Additional file 3. Participant consent form. Participant information and consent form, as approved by the Research Ethics Committee of Southern Denmark – in Danish and English translation. [file 13063_2024_8079_MOESM3_ESM.docx]

**DEPRE-ST - A randomized controlled trial of schema therapy for patients with chronic treatment resistant depression**

(Danish title: DEPRE-ST – en randomiseret, kontrolleret undersøgelse af schematerapi for patienter med svært behandlelig depression)

We would like to ask if you would like to participate in a scientific study being carried out by PhD student and psychologist Ida-Marie Arendt and psychologist, associate professor and research leader Stine Bjerrum Moeller at the psychiatric center in Region of Southern Denmark and Capital Region Psychiatry. The study is affiliated with the University of Southern Denmark.

Before deciding whether to participate in the trial, you must fully understand what the trial is about and why we are conducting the trial. We would therefore ask you to read this participant information carefully. You will also get the information further elaborated before the first assessment, where you can ask any questions you have about the study. You are welcome to bring a family member, friend or acquaintance to the interview.

If you decide to participate in the trial, we will ask you to sign a consent form. Remember that you have the right to 24 hours of reflection before you decide whether you want to sign the declaration of consent.

Participation in the study is voluntary. You can withdraw your consent at any time and without giving a reason by contacting the project manager, PhD student Ida-Marie Arendt, phone number 24658907, email: imarendt@health.sdu.dk, in writing or by phone. If you choose not to participate, or withdraw at a later stage, this will have no consequences for your further treatment in psychiatry. If you choose to withdraw after you have enrolled in the study, we will no longer contact you for follow-up measurements. However, we would like to be allowed to contact you one last time to hear your reasons for withdrawing, as this is very valuable information for the study.

**Purpose of the study**

In DEPRE-ST, we will investigate the effect of 30 sessions of individual treatment with the psychotherapy schema therapy compared to the treatment otherwise given in psychiatric settings in Denmark (e.g., group or individual treatment with other types of therapy). More precisely, we will look at the effect on difficult-to-treat depression, which is when depression has lasted more than 2 years or has been treated with more than 2 types of antidepressant medication without a sufficiently good effect.

Schema therapy is a particularly in-depth and vivid form of therapy that has shown promising results in the treatment of depression, and we will now investigate the therapy's effect more systematically.

We aim to recruit 129 participants who have been referred for depression treatment in psychiatry. You will be randomly assigned (through so-called randomization) as to whether you receive the usual psychiatric treatment or individual schema therapy. If you are assigned schema therapy, you still have the option of also receiving the treatment you would otherwise have received in psychiatry, e.g. with medication - it is only the psychotherapeutic treatment that is different.

We will also develop a model for understanding patients with difficult-to-treat depression, so that treatment providers can map out the nuances of problems in benefiting from the treatment and thus provide the right help.

**Plan for the study**

If you agree to participate in the study, we would like to make repeated measurements of your symptoms and your well-being. This is done by a short interview about your symptoms of depression and then filling in a series of questionnaires. The first assessment is expected to last approximately 1½ hours in total. All measurements take place either at the psychiatric center or online and are carried out by trained psychologists. We make measurements at enrollment in the project and again after 6 months, as well as 1 and 2 years thereafter, as it is important to assess the effect of the treatment over a longer period of time.

We measure: symptoms of depression, daily level of functioning, psychological well-being, anger management, personal assumptions about anger, anxiety symptoms, repeated negative thinking, personal recovery (perceived coping with life) after mental illness, labor market attachment, health-related quality of life, expectations for recovery from depression, and self-defined mental recovery. In addition, the first examination will uncover any previous depressions and treatments for depression, as well as education, marital status, schemas and schema modes (psychological phenomena that may underlie the depression) and whether there has been trauma in childhood. Experienced negative effects from the treatment are also measured.

It is important that you can set aside time to participate in all measurements. We agree on times for assessment according to your schedule. It is possible to meet online if you prefer.

The treatment itself takes place at the psychiatric center you are referred to, by the center's employed therapists. Treatment sessions will be recorded on video and used to internally assess the content and quality of the treatment.

**Benefits of the study**

Your participation will help in the attempt to further develop the treatment for depression in psychiatry. If schema therapy proves to be better than the treatment currently offered by psychiatry, this could be used in the planning of future psychiatric treatment. The study also provides important and valuable knowledge about the special group of patients with long-term or difficult-to-treat depression. This knowledge will potentially be used in research and treatment of depression worldwide.

If you are assigned schema therapy, you will receive individual psychotherapy adapted to you. Up to 30 sessions of schema therapy are given, which is more than is usually offered in psychiatry. Schema therapy is also a lively and engaging form of therapy that delves into emotional problems that can go all the way back to childhood.

No money or gifts are given for your participation in the study.

**Side effects, risks, complications and disadvantages**

We do not expect schema therapy to have other side effects than those already known in psychotherapy - for example temporary worsening of the condition when working with difficult emotional material. Only few people experience a permanent worsening due to psychotherapy itself.

Some people also experience being emotionally affected when they have to participate in research interviews and fill in questionnaires. If this happens, our experienced research staff will take care of you in the best possible way. You are also encouraged to talk to your therapist along the way if you experience getting worse, so you can deal with this together. The therapists who provide schema therapy receive frequent guidance (supervision) so that they can give you the best possible treatment.

However, there may be risks with the study that we do not yet know. We therefore ask you to let us know if you experience problems with your health while the trial is ongoing. If we discover side effects from the treatment that we have not already told you about, you will of course be informed immediately, and you will decide whether you want to continue with the trial.

**Exclusion from and suspension of trial**

There may be circumstances where we may need to discontinue your participation in the trial. This can be if your treatment is transferred to another treatment unit, for example if during the course you are in acute danger of suicide, if you develop mania or psychosis or are admitted to a psychiatric ward for a long period of time. The trial as a whole can be interrupted if - contrary to expectations - we find that there are previously unknown side effects from the treatment.

**Access to electronic patient records**

If you have given your consent after the initial interview at your psychiatric centre, the clinician will pass on your name, telephone number and status of depression treatment to the researchers. If you wish to participate in the study, we will also ask for your consent to direct access to your electronic patient record for, e.g., to be able to see what psychiatric treatment you have received before and during the study as well as measurements regarding your mental health. This is to be able to register and measure which treatment elements you have received and how they have worked, and also to keep an eye on the quality of the treatment. For more information, see below in the section: Information on the processing of personal data.

**Access to trial results**

The results of the trial will - in anonymized form - be made public on an ongoing basis in international journals and at scientific conferences, for the benefit of researchers and practitioners of mental illness. Relevant interest associations will also be involved in the project and its results.

The project is expected to be completed in January 2028. You have the opportunity to obtain information about the overall results of the study yourself when it has been completed. If you wish this, you can tick the attached declaration of consent.

**Information about financial conditions**

The study is financially supported with approx. DKK 6 million from Trygfonden, a private foundation which is independent of the study's initiators, Ida-Marie Arendt and Stine Bjerrum Moeller. Neither Trygfonden nor the initiators of the study have financial interests in the results of the study. In addition, the study is supported with DKK 592,000 from the Region of Southern Denmark's PhD fund.

The money is spent on PhD salary, salary for the study's research assistants, training and supervision of the participating clinicians, salary for statisticians, as well as overhead and PhD tuition fee to the University of Southern Denmark.

**Information on the processing of personal data**

In connection with the project 'DEPRE-ST - a randomized, controlled study of schema therapy for patients with chronic, difficult-to-treat depression', the Region of Southern Denmark would like to collect information about you. The Region of Southern Denmark is responsible for the protection of your personal data for use in research projects.

The collection takes place via clinical interviews and questionnaires (as described above) in connection with treatment for depression at the Region of Southern Denmark and the Capital Region of Denmark Psychiatry.

The project requests direct access to personal and health information in your electronic patient record in order to be able to assess your effect of treatment, as well as which and how much treatment you have received, both in connection to and within the project (talk therapy, medicine, etc.). In addition, access may be needed for quality checks by the project's researchers and research assistants and for monitoring of the study.

**Purpose of the processing of personal data**

The information that is processed is:

- Health information that appears from patient records regarding treatment for depression in psychiatry.

- Clinical interviews and questionnaires with a focus on mental health and psychological symptoms.

- Information on the reason for termination of treatment before originally planned, in the event of this

- Video recordings of your treatment sessions

- In addition, the researcher will be familiar with the name, e-mail address, social security number, number of attendances and medication in the psychiatric treatment as well as marital status, education and connection to the labor market.

**How Region Southern Denmark handles the personal data**

The information is processed in accordance with Section 10 of the Data Protection Act for sensitive personal data (e.g. diagnosis and health status) and in accordance with Article 6, paragraph 1, letter e of the Data Protection Regulation for the general personal data (e.g. name and email address). The Region of Southern Denmark will treat the personal data confidentially - in accordance with applicable law.

We make sure to store the data safely. The information is analyzed by our statisticians in Denmark and Austria, with whom we have entered into a formal cooperation agreement. The information will only be used for research. However, to the extent that it is relevant, the information will be recorded, cf. the Danish rules for record-keeping.

**Deletion and storage of your personal data**

Region Southern Denmark will delete or anonymize data when it is no longer relevant to store your personal data. This will most often be when the project has been completed, but can also be later, for reasons of possible documentation of research results. This will happen no later than five years after the end of the project, i.e. no later than 31-1-2033.

You should be aware:

• that you can always revoke your consent to participate in the project. Note that revoking your consent to participate will not cause information already collected to be deleted.

• that you have the right to complain to the Data Protection Authority about the processing of the information via www.datatilsynet.dk.

There will be no publication of data where you can be identified, as all data published as research will be anonymized.

**Additional information about personal data**

If you have questions about data protection and your rights, you can contact the Data Protection Adviser of the Region of Southern Denmark on telephone number +45 24 75 62 90 (phone hours Mondays and Thursdays at 9-11 AM) or by email: databeskyttelsesraadgiver@rsyd.dk

You can also read more about our processing of your personal data and your rights here: https://regionsyddanmark.dk/om-region-syddanmark/sadan-behandler-vi-dine-data-og-personoplysninger/persondatapolitik-for-region-syddanmark

-------------------------------------------------- -----------------

We hope that with this information you have gained sufficient insight into what it means to participate in the trial and that you feel equipped to make the decision about your possible participation. We also ask you to read the attached material, "Participants’ rights in a health science research project".

If you want to know more about the trial, you are very welcome to contact the project leader, PhD- student Ida-Marie T. P. Arendt, tel. 24658907, e-mail: imarendt@health.sdu.dk

With best regards

Ida-Marie T. P. Arendt, PhD, cand.psych.aut.

University of Southern Denmark, Department of Psychology, Campusvej 55, 5230 Odense/Region of Southern Denmark Psychiatry, Afdeling for Traume- og Torturoverlevere, Vestre Engvej 51, Indg. B, 3. sal, 7100 Vejle, Denmark

**Informed consent for participation in a health science research project**

*Title of the research project: 'DEPRE-ST - a randomized, controlled study of schema therapy for patients with chronic treatment resistant depression'*

Statement from the subject:

- I have received written and oral information, and I know enough about the purpose, method, benefits and disadvantages in order to be able to agree to participate.
- I understand that participation is voluntary and that I can always withdraw my consent to participate without losing my current or future rights to treatment.
- I consent to participate in the research project and have received a copy of this consent form as well as a copy of the written information about the project for my own use.
- I understand that information about me will only be used for research.
- I also give consent for information to be obtained from my electronic patient record as described in the document 'Information on processing of personal data'.

Mark with x for consent to the above*: __________*

I am informed that I can withdraw my consent to participate in the project at any time by contacting the project manager Ida-Marie T. P. Arendt, e-mail: imarendt@health.sdu.dk, telephone +4524658907.

Name of participant: ________________________________________________________

Date: _______________ Signature: ____________________________________________

If new significant health information about you arises during the research project, you will be informed. If you would *not* to receive information about new important health information that arises during the research project, please tick here: __________ (mark with an x)

Do you wish to be informed about the results of the research project and any consequences for you?

Yes _____ (mark with an x) No _____ (mark with an x)

**Declaration by the person providing the information:**

I declare that the subject has received oral and written information about the study.

I confirm that sufficient information has been provided for a decision to be made regarding participation in the trial.

The name of the person providing the information: Ida-Marie T. P. Arendt

Date: _______________ Signature: ____________________________________________

**DEPRE-ST – en randomiseret, kontrolleret undersøgelse af schematerapi for patienter med svært behandlelig depression**

(originaltitel: DEPRE-ST - A randomized controlled trial of schema therapy for patients with chronic treatment resistant depression

Vi vil spørge, om du vil deltage i et videnskabeligt forsøg, der udføres af ph.d.-studerende og psykolog Ida-Marie Arendt og psykolog, lektor og forskningsleder Stine Bjerrum Møller på psykiatriske centre i Region Syddanmark og Region Hovedstaden Psykiatri. Studiet er tilknyttet Syddansk Universitet.

Før du beslutter, om du vil deltage i forsøget, skal du fuldt ud forstå, hvad forsøget går ud på, og hvorfor vi gennemfører forsøget. Vi vil derfor bede dig om at læse denne deltagerinformation grundigt. Du vil desuden få informationerne yderligere uddybet ved en samtale, hvor du kan stille de spørgsmål, du har om forsøget. Du er velkommen til at tage et familiemedlem, en ven eller en bekendt med til samtalen.

Hvis du beslutter dig for at deltage i forsøget, vil vi bede dig om at underskrive en samtykkeerklæring. Husk, at du har ret til 24 timers betænkningstid, før du beslutter, om du vil underskrive samtykkeerklæringen.

Det er frivilligt at deltage i forsøget. Du kan når som helst og uden at give en grund trække dit samtykke tilbage ved skriftlig eller mundtlig henvendelse til projektleder, ph.d.-studerende Ida-Marie Arendt, tlf.nr.24658907, mail: [imarendt@health.sdu.dk](mailto:imarendt@health.sdu.dk). Hvis du vælger ikke at deltage eller trækker dig på et senere tidspunkt, vil det ikke få konsekvenser for din videre behandling i psykiatrien. Hvis du vælger at trække dig, efter du er startet i studiet, vil vi ikke længere indkalde dig til opfølgende målinger. Vi vil dog gerne have lov at kontakte dig en sidste gang for at høre dine årsager til at trække dig, da dette er meget værdifuld information for studiet.

**Formål med studiet**

Vi vil i DEPRE-ST undersøge effekten af 30 sessioners individuel behandling med psykoterapien schematerapi i forhold til den behandling, der ellers gives i psykiatrien (fx gruppe- eller individuel behandling med andre typer af terapi). Mere præcist vil vi se på effekten på *svært behandlelig depression*, som er når depression har varet mere end 2 år eller er blevet behandlet med mere end 2 slags antidepressiv medicin uden tilstrækkelig god effekt.

Schematerapi er en særligt dybdegående og levende terapiform, som har vist lovende resultater i behandling af depression, og vi vil nu undersøge terapiens virkning mere systematisk.

Vi vil gerne rekruttere 129 patienter, som er henvist til depressionsbehandling i psykiatrien. Det bliver tilfældigt tildelt (gennem såkaldt randomisering), om du modtager den sædvanlige psykiatriske behandling eller individuel schematerapi. Hvis du bliver tildelt schematerapi, har du stadig mulighed for også at få den behandling, du ellers ville have fået i psykiatrien, fx med medicin – det er kun den psykoterapeutiske behandling, der er anderledes.

Vi vil desuden udvikle en måde at forstå patienter med svært behandlelig depression, så man bedre kan se nuancerne i deres problemer med at få udbytte af behandlingen og dermed give den rigtige hjælp.

**Plan for forsøget**

Hvis du siger ja til at deltage i studiet, vil vi gerne lave løbende målinger af dine symptomer og dit velbefindende. Dét sker ved et kort interview omkring dine depressionssymptomer og derefter udfyldelse af en række spørgeskemaer. Første måling forventes at vare i alt ca 1½ time. Alle målinger foregår enten på det psykiatriske center eller online og foretages af uddannede psykologer. Vi laver målinger ved optag i projektet og igen efter 6 måneder, samt 1 og 2 år herefter, da det er vigtigt at vurdere effekten af behandlingen over længere tid.

Vi måler på: depressionssymptomer, dagligt funktionsniveau, psykisk velbefindende, vredeshåndtering, personlige antagelser om vrede, angstsymptomer, gentagen negativ grublen, personlig recovery (oplevet livsmestring) efter psykisk sygdom, arbejdsmarkedstilknytning, helbredsrelateret livskvalitet, forventninger til depressionens bedring og selvdefineret psykisk bedring. Desuden vil den første undersøgelse afdække eventuelle tidligere depressioner og behandlinger herfor, uddannelse, civilstatus, schemaer og schema modes (psykiske fænomener, der kan ligge til baggrund for depressionen) samt om der har været traumer i barndommen. Efter endt behandling måles på evt. oplevede negative effekter ved behandlingen.

Det er vigtigt, at du kan sætte tid af til at deltage i alle målinger. Tidspunkterne aftaler vi løbende. Der er mulighed for at mødes online, hvis du skulle foretrække det.

Selve behandlingen foregår på det psykiatriske center, du er henvist til, ved centrets ansatte terapeuter. Alle behandlingssessioner vil blive optagede på video og anvendt til internt at vurdere indhold og kvalitet af behandlingen.

**Nytte ved forsøget**

Din deltagelse vil hjælpe i et forsøg på at videreudvikle behandlingen for depression i psykiatrien. Hvis schematerapi viser sig at være bedre end den behandling, psykiatrien tilbyder i øjeblikket, vil dette kunne bruges i planlægningen af fremtidig psykiatrisk behandling. Studiet skaffer desuden vigtig og værdifuld viden om den særlige gruppe af patienter med langvarig eller svært behandlelig depression. Denne viden vil potentielt kunne bruges i forskning og behandling af depression over hele verden.

Hvis du får tildelt schematerapi som behandlingsform, får du individuel psykoterapi tilpasset dig. Der gives op til 30 sessioners schematerapi, hvilket er flere, end der sædvanligvis tilbydes i psykiatrien. Schematerapi er desuden en levende og engagerende terapiform, som går i dybden med følelsesmæssige problematikker, der kan række helt tilbage til barndommen.

Der gives ikke penge eller gaver for din deltagelse i forsøget.

**Bivirkninger, risici, komplikationer og ulemper**

Vi forventer ikke, at schematerapi har andre mulige bivirkninger end dem, der allerede er kendte ved psykoterapi - fx forbigående forværring af tilstanden, når der arbejdes med svært følelsesmæssige materiale. Kun få patienter oplever en blivende forværring på grund af psykoterapi i sig selv.

Nogle patienter oplever desuden at blive følelsesmæssigt påvirket, når de skal deltage i forskningsinterviews og udfylde spørgeskemaer. Sker dette, vil vores erfarne forskningsmedarbejdere tage hånd om dig på bedste vis. Du opfordres desuden til at tale med din behandler undervejs, hvis du oplever at få det værre, så I sammen kan hjælpe dig videre. Behandlerne, der giver schematerapi, modtager hyppig vejledning (supervision), så de kan give dig den bedst mulige behandling.

Der kan dog være risici ved forsøget, som vi endnu ikke kender. Vi beder dig derfor om at fortælle, hvis du oplever problemer med dit helbred, mens forsøget står på. Hvis vi opdager bivirkninger ved behandlingen, som vi ikke allerede har fortalt dig om, vil du naturligvis blive orienteret med det samme, og du vil skulle tage stilling til, om du ønsker at fortsætte i forsøget.

**Udelukkelse fra og afbrydelse af forsøg**

Der kan være omstændigheder, hvor vi kan være nødt til at afbryde din deltagelse i forsøget. Dette kan være, hvis behandlingen overgår i andet regi, fx hvis du under forløbet kommer i akut selvmordsfare, hvis du udvikler mani eller psykose eller bliver indlagt længerevarende på en psykiatrisk afdeling. Forsøget som helhed kan afbrydes, hvis vi - mod forventning - finder, at der er hidtil ukendte bivirkninger ved behandlingen.

**Adgang til elektronisk patientjournal**

Hvis du har givet samtykke til det efter den indledende samtale på dit psykiatriske center, videregiver klinikeren navn, telefonnummer og status på depressionsbehandling til forskerne. Hvis du ønsker at deltage i studiet, vil vi også bede om dit samtykke til direkte adgang til din elektroniske patientjournal for bl.a. at kunne se, hvilken psykiatrisk behandling, du har fået før og under studiet samt målinger angående dit psykiske helbred. Dette er for at kunne registrere og måle på, hvilke behandlingselementer du har fået og hvordan de har virket, og desuden for at holde øje med kvaliteten af behandlingen. For mere information, se nedenfor i afsnittet: Oplysning om behandling af personoplysninger.

**Adgang til forsøgsresultater**

Forsøgets resultater vil – i anonymiseret form - løbende blive offentliggjort i internationale tidsskrifter og på videnskabelige konferencer, til gavn for forskere og behandlere af psykisk sygdom. Relevante interesseforeninger vil desuden blive delagtiggjort i projektet og dets resultater.

Projektet forventes afsluttet i januar 2028. Du har mulighed for at selv at få oplysninger om studiets samlede resultater, når det er udført. Hvis du ønsker dette, kan du sætte kryds på den vedlagte samtykkeerklæring.

**Oplysninger om økonomiske forhold**

Studiet er støttet økonomisk med ca. 6. mio kr af Trygfonden, som er uafhængig af studiets initiativtagere, Ida-Marie Arendt og Stine Bjerrum Møller. Hverken Trygfonden eller studiets initiativtagere har økonomiske interesser i studiets resultater. Desuden er studiet støttet med 592000 kr fra Region Syddanmarks ph.d.-pulje.

Støtten går bl.a. til ph.d.- løn, løn til studiets forskningsassistenter, uddannelse og supervision af de medvirkende klinikere, løn til statistiker, samt overhead og ph. d.-afgift til Syddansk Universitet.

**Oplysning om behandling af personoplysninger**

I forbindelse med projektet ’DEPRE-ST – en randomiseret, kontrolleret undersøgelse af schematerapi for patienter med kronisk, svært behandlelig depression’ ønsker Region Syddanmark at indsamle oplysninger om dig. Region Syddanmark er ansvarlig for beskyttelsen af dine personoplysninger til brug for forskningsprojekter.

Indsamlingen sker via kliniske interviews og spørgeskemaer (som beskrevet ovenfor) i forbindelse med behandling for depression ved Region Syddanmark og Region Hovedstaden Psykiatri.

Der er behov for at få direkte adgang til person- og helbredsoplysninger i din elektroniske patientjournal for at kunne vurdere din respons på behandlingen samt hvilken og hvor meget behandling, du har modtaget, både i forbindelse med og inden projektet (samtaleterapi, medicin mv). Desuden kan der være brug for adgang i forbindelse med kvalitetstjek af projektets forskere og forskningsassistenter eller overvågning og kontrol af forsøget.

**Formål med behandlingen af personoplysninger**

De oplysninger, der behandles er:

- Helbredsoplysninger, som fremgår af patientjournaler ifm behandling for depression i psykiatrien.
- Kliniske interviews og spørgeskemaer med fokus på mentalt helbred og psykiske symptomer.
- Ved ophør med behandling før oprindeligt planlagt, oplysninger om årsag hertil
- Videooptagelser af alle dine behandlingssessioner
- Derudover vil den forsøgsansvarlige være bekendt med navn, e-mailadresse, cpr-nr., antallet af fremmøder og medicinering i den psykiatriske behandling samt civilstatus, uddannelse og tilknytning til arbejdsmarkedet.

**Sådan bruger Region Syddanmark personoplysningerne**

Oplysningerne behandles efter databeskyttelseslovens § 10 for de følsomme personoplysninger (fx diagnose og helbredsstatus) og efter databeskyttelsesforordningens artikel 6, stk. 1, litra e for de almindelige personoplysninger (fx navn og mail-adresse). Region Syddanmark vil behandle personoplysningerne fortroligt - i overensstemmelse med gældende ret.

Vi sørger for at opbevare dem sikkert. Oplysningerne analyseres af vores statistikere i Danmark og Østrig, som vi har indgået en formel samarbejdsaftale med. Oplysningerne vil alene blive brugt til forskning. Dog vil oplysningerne i det omfang, det er relevant, blive journaliseret, jf. journalføringsbekendtgørelsens regler.

**Sletning og opbevaring af dine personoplysninger**

Region Syddanmark vil slette eller anonymisere data, når det ikke længere er relevant at opbevare dine personoplysninger. Det vil oftest være når projektet er afsluttet, men kan også være senere, af hensyn til eventuel dokumentation for forskningsresultater. Det vil ske senest fem år efter projektets afslutning, dvs. senest d. 31-1-2033.

Du gøres opmærksom på,

- at du altid kan tilbagekalde dit samtykke til deltagelse i projektet. Bemærk, at en tilbagekaldelse af dit samtykke til deltagelse ikke vil bevirke, at oplysninger, der allerede er indsamlet, bliver slettet.
- at du har ret til at klage til Datatilsynet over behandlingen af oplysningerne via

www.datatilsynet.dk.

Der vil ikke ske offentliggørelse af data, hvor du kan identificeres, da al data, der publiceres som forskning bliver anonymiseret.

**Yderligere information om persondata**

Hvis du har spørgsmål omkring databeskyttelse og dine rettigheder kan du kontakte Region Syddanmarks databeskyttelsesrådgiver på tlf. 24 75 62 90 (telefontid mandag og torsdag kl. 9-11) eller mail: databeskyttelsesraadgiver@rsyd.dk

Du kan også læse mere om vores behandling af dine personoplysninger og dine rettigheder hér: <https://regionsyddanmark.dk/om-region-syddanmark/sadan-behandler-vi-dine-data-og-personoplysninger/persondatapolitik-for-region-syddanmark>

-------------------------------------------------------------------

Vi håber, at du med denne information har fået tilstrækkeligt indblik i, hvad det vil sige at deltage i forsøget, og at du føler dig rustet til at tage beslutningen om din eventuelle deltagelse. Vi beder dig også om at læse det vedlagte materiale, ”Forsøgspersonens rettigheder i et sundhedsvidenskabeligt forskningsprojekt”.

Hvis du vil vide mere om forsøget, er du meget velkommen til at kontakte projektleder, ph.d.-studerende Ida-Marie T. P. Arendt, tlf 24658907, e-mail: [imarendt@health.sdu.dk](mailto:imarendt@health.sdu.dk).

Med venlig hilsen

Ida-Marie T. P. Arendt, ph.d.stud., cand.psych.aut.

Syddansk Universitet, Institut for Psykologi, Campusvej 55, 5230 Odense/Region Syddanmark Psykiatri, Afdeling for Traume- og Torturoverlevere, Vestre Engvej 51, Indg. B, 3. sal, 7100 Vejle

**Informeret samtykke til deltagelse i sundhedsvidenskabeligt forskningsprojekt**

*Forskningsprojektets titel: ’DEPRE-ST – en randomiseret, kontrolleret undersøgelse af schematerapi for patienter med svært behandlelig depression’*

**Erklæring fra forsøgspersonen**:

Jeg har fået skriftlig og mundtlig information, og jeg ved nok om formål, metode, fordele og
ulemper til at sige ja til at deltage.

Jeg ved, at det er frivilligt at deltage, og at jeg altid kan trække mit samtykke til deltagelse tilbage uden at miste mine nuværende eller fremtidige rettigheder til behandling.

Jeg giver samtykke til at deltage i forskningsprojektet og har fået en kopi af dette samtykkeark
samt en kopi af den skriftlige information om projektet til eget brug.

Jeg forstår, at oplysninger om mig kun vil blive anvendt til forskning.

Jeg giver desuden samtykke til, at der indhentes oplysninger fra min elektroniske patientjournal som beskrevet i dokumentet ’Oplysning om behandling af personoplysninger’.

Sæt kryds ved samtykke hertil: __________

Jeg er orienteret om, at jeg til hver en tid kan trække mit samtykke til deltagelse i projektet tilbage ved at kontakte projektleder Ida-Marie T. P. Arendt, e-mail: imarendt@health.sdu.dk - tlf 24658907.

Forsøgspersonens navn: ________________________________________________________

Dato: _______________ Underskrift: ____________________________________________

Hvis der kommer nye væsentlige helbredsoplysninger frem om dig i forskningsprojektet vil du blive informeret. Vil du **frabede** dig information om nye væsentlige helbredsoplysninger, som kommer frem i forskningsprojektet, bedes du markere her: __________ (sæt x)

Ønsker du at blive informeret om forskningsprojektets resultat samt eventuelle konsekvenser for dig?:

Ja _____ (sæt x) Nej _____ (sæt x)

**Erklæring fra den, der afgiver information:**

Jeg erklærer, at forsøgspersonen har modtaget mundtlig og skriftlig information om forsøget.

Efter min overbevisning er der givet tilstrækkelig information til, at der kan træffes beslutning om deltagelse i forsøget.

Navnet på den, der afgiver information: Ida-Marie T. P. Arendt

Dato: _______________ Underskrift: ____________________________________________
