# Supplementary material for: Schema therapy versus treatment as usual for outpatients with difficult-to-treat depression: study protocol for a parallel group randomized clinical trial (DEPRE-ST)
Source: Trials. 2024 Apr 16;25:266. doi: 10.1186/s13063-024-08079-9 (PMC11022394; doi:10.1186/s13063-024-08079-9)
Supplement: Supplementary file 4 — Additional file 4. Details about randomization. [file 13063_2024_8079_MOESM4_ESM.docx]

# Randomization generation:

A randomization list with permuted block sizes of 2, 4, and 6 was generated by Sealed Envelope Ltd. 2022. at https://sealedenvelope.com/simple-randomiser/v1/lists Create a blocked randomisation list. [Online] [Accessed 10 Feb 2023].

The randomization list is uploaded to REDCap which provides the stratified randomization.
