# Supplementary material for: Schema therapy versus treatment as usual for outpatients with difficult-to-treat depression: study protocol for a parallel group randomized clinical trial (DEPRE-ST)
Source: Trials. 2024 Apr 16;25:266. doi: 10.1186/s13063-024-08079-9 (PMC11022394; doi:10.1186/s13063-024-08079-9)
Supplement: Supplementary file 5 — Additional file 5. Outcome forms. Outcomes (with the exception of forms with copyright, i.e. Childhood Trauma Questionnaire, Symptom Checklist-5, Young Schema Questionnaire, Schema Mode Inventory). [file 13063_2024_8079_MOESM5_ESM.pdf]

Additional file 5 - *Outcome forms*

Primary outcome

HAMD-6:

DEPRESSED MOOD *(sadness, hopeless, helpless, worthless)*

0 |__| Absent.

1 |__| These feeling states indicated only on questioning.

2 |__| These feeling states spontaneously reported verbally.

3 |__| Communicates feeling states non-verbally, i.e. through facial expression, posture, voice and tendency to weep.

4 |__| Patient reports virtually only these feeling states in his/her spontaneous verbal and non-verbal communication.

FEELINGS OF GUILT

0 |__| Absent.

1 |__| Self reproach, feels he/she has let people down.

2 |__| Ideas of guilt or rumination over past errors or sinful deeds.

3 |__| Present illness is a punishment. Delusions of guilt.

4 |__| Hears accusatory or denunciatory voices and/or experiences threatening visual hallucinations.

WORK AND ACTIVITIES

0 |__| No difficulty.

1 |__| Thoughts and feelings of incapacity, fatigue or weakness related to activities, work or hobbies.

2 |__| Loss of interest in activity, hobbies or work – either directly reported by the patient or indirect in listlessness, indecision and vacillation (feels he/she has to push self to work or activities).

3 |__| Decrease in actual time spent in activities or decrease in productivity. Rate 3 if the patient does not spend at least three hours a day in activities (job or hobbies) excluding routine chores.

4 |__| Stopped working because of present illness. Rate 4 if patient engages in no activities except routine chores, or if patient fails to perform routine chores unassisted.

ANXIETY PSYCHIC

0 |__| No difficulty.

1 |__| Subjective tension and irritability.

2 |__| Worrying about minor matters.

3 |__| Apprehensive attitude apparent in face or speech.

4 |__| Fears expressed without questioning.

GENERAL SOMATIC SYMPTOMS

0 |__| None.

1 |__| Heaviness in limbs, back or head. Backaches, headaches, muscle aches. Loss of energy and fatigability.

2 |__| Any clear-cut symptom rates 2.

8 RETARDATION (slowness of thought and speech, impaired ability to concentrate, decreased motor activity)

0 |__| Normal speech and thought.

1 |__| Slight retardation during the interview.

2 |__| Obvious retardation during the interview.

3 |__| Interview difficult.

4 |__| Complete stupor.

Secondary outcomes:

European Quality of Life 5 Dimensions 5 Level Version

MOBILITY

I have no problems in walking about ☐
I have slight problems in walking about ☐
I have moderate problems in walking about ☐
I have severe problems in walking about ☐
I am unable to walk about ☐

SELF-CARE

I have no problems washing or dressing myself ☐
I have slight problems washing or dressing myself ☐
I have moderate problems washing or dressing myself ☐
I have severe problems washing or dressing myself ☐
I am unable to wash or dress myself ☐

USUAL ACTIVITIES *(e.g. work, study, housework, family or leisure activities)*

I have no problems doing my usual activities ☐
I have slight problems doing my usual activities ☐
I have moderate problems doing my usual activities ☐
I have severe problems doing my usual activities ☐
I am unable to do my usual activities ☐

PAIN / DISCOMFORT

I have no pain or discomfort ☐
I have slight pain or discomfort ☐
I have moderate pain or discomfort ☐
I have severe pain or discomfort ☐
I have extreme pain or discomfort ☐

ANXIETY / DEPRESSION

I am not anxious or depressed ☐
I am slightly anxious or depressed ☐
I am moderately anxious or depressed ☐
I am severely anxious or depressed ☐
I am extremely anxious or depressed ☐

Work and Social Adjustment Scale (WSAS).

		Not at All		Slightly		Definitely		Markedly		Very Severely
1.	Because of my mental health my **ability to work** is impaired. '0' means 'not at all impaired' and '8' means very severely impaired to the point I can't work.	0	1	2	3	4	5	6	7	8
2.	Because of my mental health my **home management** (cleaning, tidying, shopping, cooking, looking after home or children, paying bills) is impaired.	0	1	2	3	4	5	6	7	8
3.	Because of my mental health my **social leisure activities** (with other people e.g. parties, bars, clubs, outings, visits, dating, home entertaining) are impaired.	0	1	2	3	4	5	6	7	8
4.	Because of my mental health, my **private leisure activities** (done alone, such as reading, gardening, collecting, sewing, walking alone) are impaired.	0	1	2	3	4	5	6	7	8
5.	Because of my mental health, my ability to form and maintain **close relationships** with others, including those I live with, is impaired.	0	1	2	3	4	5	6	7	8

WHO-5 Well-Being Index.

Over the last two weeks:	All the time	Most of the time	More than half of the time	Less than half of the time	Some of the time	At no time
**1. I have felt cheerful and in good spirits**	5	4	3	2	1	0
**2. I have felt calm and relaxed**	5	4	3	2	1	0
**3. I have felt active and vigorous**	5	4	3	2	1	0
**4. I woke up feeling fresh and rested**	5	4	3	2	1	0
**5. My daily life has been filled with things that interest me**	5	4	3	2	1	0

Negative Effects Questionnaire (NEQ).

	Incidents and effects:	Did you experience this?		*If yes* – here is how negatively it affected me:					Probably caused by:	
		No	Yes	Not at all	Slightly	Moderately	Very	Extremely	The treatment I received	Other circumstances
1.	I had more problems with my sleep	○	○➔	○	○	○	○	○	○	○
2.	I felt like I was under more stress	○	○➔	○	○	○	○	○	○	○
3.	I experienced more anxiety	○	○➔	○	○	○	○	○	○	○
4.	I felt more worried	○	○➔	○	○	○	○	○	○	○
5.	I experienced more hopelessness	○	○➔	○	○	○	○	○	○	○
6.	I experienced more unpleasant feelings	○	○➔	○	○	○	○	○	○	○
7.	I felt that the issue I was looking for help with got worse	○	○➔	○	○	○	○	○	○	○
8.	Unpleasant memories resurfaced	○	○➔	○	○	○	○	○	○	○
9.	I became afraid that other people would find out about my treatment	○	○➔	○	○	○	○	○	○	○
10.	I got thoughts that it would be better if I did not exist anymore and that I should take my own life	○	○➔	○	○	○	○	○	○	○
11.	I started feeling ashamed in front of other people because I was having treatment	○	○➔	○	○	○	○	○	○	○
12.	I stopped thinking that things could get better	○	○➔	○	○	○	○	○	○	○
13.	I started thinking that the issue I was seeking help for could not be made any better	○	○➔	○	○	○	○	○	○	○
14.	I think that I have developed a dependency on my treatment	○	○➔	○	○	○	○	○	○	○
15.	I did not always understand my treatment	○	○➔	○	○	○	○	○	○	○
16.	I did not always understand my therapist	○	○➔	○	○	○	○	○	○	○
17.	I did not have confidence in my treatment	○	○➔	○	○	○	○	○	○	○
18.	I felt that the treatment did not produce any results	○	○➔	○	○	○	○	○	○	○
19.	I felt that my expectations for the therapist were not fulfilled	○	○➔	○	○	○	○	○	○	○
20.	I felt that the treatment was not motivating	○	○➔	○	○	○	○	○	○	○

Other incidents or effects – describe in your own words whether there were any other negative incidents or effects, and what characterised them	

Exploratory outcomes:

Psychological Outcome Profiles (PSYCHLOPS)- pre-treatment:

Question 1

a **Choose the problem that troubles you most.** *(Please write it in the box below.)*

b **How much has it affected you over the last week?** *(Please tick one box below.)*

	0	1	2	3	4	5	
Not at all affected	☐	☐	☐	☐	☐	☐	Severely affected

c **How long ago were you first concerned about this problem?** *(Please tick one box below.)*

Under one month	Between one and three months	Over three months but under one year	One to five years	Over five years
☐	☐	☐	☐	☐

Question 2

a **Choose another problem that troubles you.** *(Please write it in the box below.)*

b **How much has it affected you over the last week?** *(Please tick one box below.)*

	0	1	2	3	4	5	
Not at all affected	☐	☐	☐	☐	☐	☐	Severely affected

c **How long ago were you first concerned about this problem?** *(Please tick one box below.)*

Under one month	Between one and three months	Over three months but under one year	One to five years	Over five years
☐	☐	☐	☐	☐

Question 3

a **Choose one thing that is hard to do because of your problem (or problems).** *(Please write it in the box below.)*

b **How hard has it been to do this thing over the last week?** *(Please tick one box below.)*

	0	1	2	3	4	5	
Not at all hard	☐	☐	☐	☐	☐	☐	Very hard

Question 4

How have you felt in yourself this last week? *(Please tick one box below.)*

	0	1	2	3	4	5	
Very good	☐	☐	☐	☐	☐	☐	Very bad

Post-treatment:

Question 1

a **This is the problem you said troubled you the most when we first asked.** *(Therapist - please write it in the box below.)*

b **How much has it affected you over the last week?** *(Please tick one box below.)*

	0	1	2	3	4	5	
Not at all affected	☐	☐	☐	☐	☐	☐	Severely affected

Question 2

a **This is the other problem you said troubled you when we first asked.** *(Therapist - please write it in the box below.)*

b **How much has it affected you over the last week?** *(Please tick one box below.)*

	0	1	2	3	4	5	
Not at all affected	☐	☐	☐	☐	☐	☐	Severely affected

Question 3

a **This is the thing you said was hard to do when we first asked.** *(Therapist - please write it in the box below.)*

b **How hard has it been to do this thing over the last week?** *(Please tick one box below.)*

	0	1	2	3	4	5	
Not at all hard	☐	☐	☐	☐	☐	☐	Very hard

Question 4

How have you felt in yourself this last week? *(Please tick one box below.)*

	0	1	2	3	4	5	
Very good	☐	☐	☐	☐	☐	☐	Very bad

Question 5

During therapy, you may have found that other problems became important. If so, how much have these problems affected you over the last week?
(Please tick one box below, or leave blank if no other problems have become important.)

	0	1	2	3	4	5	
Not at all affected	☐	☐	☐	☐	☐	☐	Severely affected

Question 6

Compared to when you started therapy, how do you feel now? *(Please tick one box below.)*

0	1	2	3	4	5
☐	☐	☐	☐	☐	☐
Much better	Quite a lot better	A little better	About the same	A little worse	Much worse

Brief INSPIRE-O.

1 I feel supported by other people	Not at all	Not much	Somewhat	Quite a lot	Very much
2 I have hopes and dreams for the future	Not at all	Not much	Somewhat	Quite a lot	Very much
3 I feel good about myself	Not at all	Not much	Somewhat	Quite a lot	Very much
4 I do things that mean something to me	Not at all	Not much	Somewhat	Quite a lot	Very much
5 I feel in control of my life	Not at all	Not much	Somewhat	Quite a lot	Very much

Dimensions of Anger Reactions – Revised (DAR)

1. I often find myself getting angry at people or situations
2. When I do get angry, I get really mad
3. When I get angry, I stay angry
4. When I get angry at someone, I want to hit or strike the person
5. My anger interferes with my ability to get my work or activities done
6. My anger prevents me from getting along with people as well as I would like to
7. My anger has a bad effect on my health

Metacognitive Anger Processing – Short Version (MAP-SV)

The statements below describe beliefs that people have about own thoughts and emotions.
How true are they for you?

For each statement please indicate whether is (1) never true, (2) sometimes true, (3) often true, (4) always true**.** Use the scale at your right to circle the answer that best describes how true the statement is for you

		Never true	Sometimes true	Often true	Always true
1	When I am angry I keep thinking about it	1	2	3	4
5	Anger could make me go mad	1	2	3	4
6	Anger helps me solve problems	1	2	3	4
7	I cannot let go of angry thoughts	1	2	3	4
9	Anger helps me handle threats and dangers	1	2	3	4
11	Anger makes me a bad person	1	2	3	4
14	My anger is dangerous for me	1	2	3	4
15	Anger makes me a strong and competent person	1	2	3	4
25	Anger stays with me for a long time	1	2	3	4

The Perseverative Thinking Questionnaire (PTQ)

In this questionnaire, you will be asked to describe how you *typically* think about negative experiences or problems. Please read the following statements and rate the extent to which they apply to you when you think about negative experiences or problems.

		Never	Rarely	Sometimes	Often	Almost always
1.	The same thoughts keep going through my mind again and again.	0	1	2	3	4
2.	Thoughts intrude into my mind.	0	1	2	3	4
3.	I can't stop dwelling on them.	0	1	2	3	4
4.	I think about many problems without solving any of them.	0	1	2	3	4
5.	I can't do anything else while thinking about my problems.	0	1	2	3	4
6.	My thoughts repeat themselves.	0	1	2	3	4
7.	Thoughts come to my mind without me wanting them to.	0	1	2	3	4
8.	I get stuck on certain issues and can't move on.	0	1	2	3	4
9.	I keep asking myself questions without finding an answer.	0	1	2	3	4
10.	My thoughts prevent me from focusing on other things.	0	1	2	3	4
11.	I keep thinking about the same issue all the time.	0	1	2	3	4
12.	Thoughts just pop into my mind.	0	1	2	3	4
13.	I feel driven to continue dwelling on the same issue.	0	1	2	3	4
14.	My thoughts are not much help to me.	0	1	2	3	4
15.	My thoughts take up all my attention.	0	1	2	3	4

TOTAL:

The Perseverative Thinking Questionnaire (PTQ): Validation of a content-independent measure of repetitive negative thinking. Available from: https://www.researchgate.net/publication/49831037_The_Perseverative_Thinking_Questionnaire_PTQ_Validation_of_a_content-independent_measure_of_repetitive_negative_thinking
