# Supplementary material for: Schema therapy versus treatment as usual for outpatients with difficult-to-treat depression: study protocol for a parallel group randomized clinical trial (DEPRE-ST)
Source: Trials. 2024 Apr 16;25:266. doi: 10.1186/s13063-024-08079-9 (PMC11022394; doi:10.1186/s13063-024-08079-9)
Supplement: Supplementary file 6 — Additional file 6. Letters of funding.Letters of funding in DKK from Trygfonden and the Region of Southern Denmark PhD-fund – Danish and English translation. [file 13063_2024_8079_MOESM6_ESM.pdf]

Ida-Marie Terese Pereira Arendt

From:	Region Southern Denmark <no-reply@efond.dk>
Late:	May 24, 2022 12:50 p.m
Thaw:	Ida-Marie Terese Pereira Arendt; mgs@rsyd.dk
Subject:	Region of Southern Denmark PhD pool 2022 - 1st announcement - A1523 Ida-Marie TP Arendt

You do not often receive emails from no-reply@efond.dk. Learn more about why this is important

This email cannot be answered directly. For questions about the content of the email, please contact forskning.sund@rsyd.dk.

Dear Ida-Marie TP Arendt

J.no.: 22/26258
Fund: 1523

Regarding your application for the Region of Southern Denmark's PhD pool 2022 with the project: DEPRE-ST: A randomized, controlled study of schema therapy for patients with chronic and treatment-resistant depression.

On 23 May 2022, the Regional Council has made a decision on which applications will receive a grant.

We are pleased to announce that your application has been approved to receive a grant from the Region of Southern Denmark Ph.d.-pool corresponding to one year's PhD scholarship worth DKK 592,000.

This email is your official grant letter and you can receive your grant from today.

For PAYMENT of your grant, we must use:

1. Your enrollment letter as a PhD student at the Faculty of Health Sciences at SDU.
2. Notification from you about where the funds are to be transferred to – either a hospital unit or SDU.
- If your funds are to be transferred to OUH, you must instead contact OUH ForskerService (reg.forskerservice@rsyd.dk)
3. Notification from you about which years the grant must be used - remember that the grant must be used no later than 4 years after you have received your official grant letter.

Your request for payment incl. enrollment letter must be sent to forskning.sund@rsyd.dk no later than one year after the date of your official grant letter!
If we have not heard from you within one year, your grant will expire.

Please note that the grant has the following requirements:
1. Accounts must be submitted when the project is completed. This is submitted to forskning.sund@rsyd.dk
2. If necessary. remaining amount must be repaid
3. Changes to the project, such as the project's period due to maternity or illness, or the project's budget, must be approved by the Department for Quality and Research, forskning.sund@rsyd.dk
4. In publications that originate from the project, it must be stated, that the Region of Southern Denmark has contributed with support.

Sincerely

The regional strategic research council
E-mail: forskning.sund@rsyd.dk

The regional house
Damhaven 12, 7100 Vejle
Main number: 7663 1000
www.rsyd.dk

Ida-Marie Terese Pereira Arendt

From: Region Syddanmark <no-reply@efond.dk>
Sent: 24. maj 2022 12:50
To: Ida-Marie Terese Pereira Arendt; mgs@rsyd.dk
Subject: Region Syddanmarks Ph.d.-pulje 2022 - 1. opslag - A1523 Ida-Marie T. P. Arendt

Du får ikke ofte mails fra no-reply@efond.dk. Få mere at vide om, hvorfor dette er vigtigt

*****Denne mail kan ikke besvares direkte. For spørgsmål til mailens indhold, da henvend dig til forskning.sund@rsyd.dk.*****

Kære Ida-Marie T. P. Arendt J.nr.:

22/26258
Efond: 1523

Vedrørende din ansøgning til Region Syddanmarks Ph.d.-pulje 2022 med projektet: DEPRE-ST: En randomiseret, kontrolleret undersøgelse af schematerapi for patienter med kronisk og behandlingsresistent depression.

Regionsrådet har d. 23. maj 2022 taget beslutning om, hvilke ansøgninger der skal modtage bevilling.

Vi kan med glæde meddele, at din ansøgning er blevet godkendt til at modtage bevilling fra Region Syddanmarks Ph.d.- pulje svarende til ét års ph.d.-stipendium á kr. 592.000.

Denne mail er dit officielle bevillingsbrev, og du kan modtage din bevilling fra dags dato.

For UDBETALING af din bevilling skal vi bruge:

1. Dit indskrivningsbrev som ph.d.-studerende på Det Sundhedsvidenskabelige Fakultet på SDU.
2. Besked fra dig om, hvor midlerne skal overføres til – enten en sygehusenhed eller SDU.

- Skal dine midler overføres til OUH, skal du i stedet kontakte OUH ForskerService (reg.forskerservice@rsyd.dk)

3. Besked fra dig om hvilke år bevillingen skal anvendes – husk, at bevillingen skal være anvendt senest 4 år efter, at du har fået dit officielle bevillingsbrev.

Din anmodning om udbetaling inkl. indskrivningsbrev skal sendes til forskning.sund@rsyd.dk senest ét år efter datoen for dit officielle bevillingsbrev!
Har vi ikke hørt fra dig indenfor ét år, vil din bevilling bortfalde.

Bemærk, at der til bevillingen er følgende krav:

1. Der skal indsendes regnskab, når projektet afsluttes. Dette indsendes til forskning.sund@rsyd.dk
2. Evt. restbeløb skal tilbagebetales
3. Ændring til projektet såsom projektets periode pga. barsel eller sygdom, eller projektets budget, skal godkendes af Afdelingen for Kvalitet og Forskning, forskning.sund@rsyd.dk
4. I publikationer, som udgår fra projektet, skal det fremgå, at Region Syddanmark har bidraget med støtte.

Venlig hilsen

Det regionale strategiske forskningsråd E-mail: forskning.sund@rsyd.dk

VS: Application (ID: 149726)

Stine Bjerrum Møller <Stine.Bjerrum.Moeller@regionh.dk>
Mon 31-08-2020 12:09

To:Ida-Marie Arendt <ida-marie.arendt@regionh.dk>

From: ansoegning@trygfonden.dk <ansoegning@trygfonden.dk>
Sent: 31 August 2020 11:04
To: Stine Bjerrum Møller <Stine. Bjerrum.Moeller@regionh.dk>
Subject: Application (ID: 149726)

TrygFonden

Dear Stine Bjerrum Møller

Thank you for your application: Schema therapy against depression (ID: 149726).

TrygFonden has processed your application, and we are happy to announce that TrygFonden will support the project with DKK 6,051,912. The amount is incl. Possibly. VAT.

As previously announced, your application has been peer reviewed. We would like to encourage you to take the peer reviewers' possible improvement suggestions under serious consideration. Unfortunately, for technical reasons, we cannot attach the ratings to this email. They can be requested by contacting Anders Hagen Hansen at aha@try.gfonden.dk.

Use of the grant

The grant is granted on the condition that the project is carried out in its entirety entirety as presented in the application. If this is not possible, a new project description and budget must be sent to TrygFonden for approval. However, this does not apply to changes in the application's project description that are prompted by the peer reviews' suggestions for improvement.

Payment

Support will be paid based on documentation of expenses, unless special circumstances apply. When you want part or all of the grant paid out, you must go to our website Tryghed.dk, click on "Log in", open the application and follow the instructions.

Support must be paid no later than one month after the end date you have calculated in the application. If this has not happened, the support will cease. If the end date for the project changes, this must be approved by TrygFonden per mail to Pia Hansen, ph@trygfonden.dk.

Disclosure

Reports, pamphlets, scientific publications, theses, articles in journals or other mention of the project must clearly state that the project has been carried out with support from TrygFonden. If you want sparring and or input into the dissemination of the project, | welcome to contact Christian Nørr, TrygFonden's documentarian, on tel. 25 45 62 26 or cn@trygfonden.dk.

In addition, please submit a copy to the foundation, with the ID number applied.

Evaluation

TrygFonden wants to collect and disseminate the results from the projects to which the foundation has donated funds. All donation recipients are therefore asked to complete an evaluation form. When your project is nearing completion, you will receive an email containing a link to an evaluation page. Please evaluate your project no later than 14 days after the end of the project.

Financial Accounting

Final accounts for the project must be submitted to TrygFonden immediately after the end of the project. The accounts must be signed by a state authorized or registered accountant. In the event that the grant only covers part of the amount applied for and the project's total budget, the accounts must still include all income and expenses for the project period.

We point out that the part of the donation that may had to cover

fees/salary expenses are taxable income.

Refund

The grant must be repaid if:

- An adequate signed account is not submitted.
- The grant or parts thereof have not been used in accordance with the conditions of the grant.

If support has been received from another party such that the project is over-financed, TrygFonden will then be able to request proportionate repayment. In the event that there is an unused amount after the project period, this must be repaid.

We wish you luck with the project and look forward to hearing more about it.

Sincerely
TrygFonden

Rie Odsbjerg Werner
Adm. Manager

This email cannot be answered.

TrygFonden smba (TryghedsGruppen smba) Hummeltoftevej 49 DK-2830 Virum
Telephone 45 26 08 00 Fax 45 26 0801. info@trygfonden.dk. safety.dk

---

This email contains confidential information. If you are not the intended recipient of this email or if you receive it in error, we apologize please inform the sender of the error using the reply function. At the same time, please delete the email immediately without forwarding or copy that.

VS: Ansøgning (ID: 149726)

Stine Bjerrum Møller <Stine.Bjerrum.Moeller@regionh.dk>
Man 31-08-2020 12:09
Til:Ida-Marie Arendt <ida-marie.arendt@regionh.dk>

Fra: ansoegning@trygfonden.dk <ansoegning@trygfonden.dk>
Sendt: 31. august 2020 11:04
Til: Stine Bjerrum Møller <Stine.Bjerrum.Moeller@regionh.dk>
Emne: Ansøgning (ID: 149726)

TrygFonden

Kære Stine Bjerrum Møller

Tak for din ansøgning: Skematerapi mod depression (ID: 149726).

TrygFonden har behandlet din ansøgning, og vi er glade for at kunne fortælle, at TrygFonden vil støtte projektet med kr. 6.051.912. Beløbet er inkl. evt. moms.

Som tidligere meddelt har din ansøgning været i peer review. Vi vil gerne opfordre dig til at tage peer reviewernes eventuelle forbedringsforslag under seriøs overvejelse. Af tekniske grunde kan vi desværre ikke vedhæfte bedømmelserne til denne mail. De kan rekvireres ved at kontakte Anders Hagen Hansen på aha@trygfonden.dk.

Anvendelse af bevillingen
Bevillingen er givet under forudsætning af, at projektet gennemføres i sin helhed som fremført i ansøgningen. Såfremt dette ikke er muligt, skal der fremsendes en ny projektbeskrivelse samt budget til TrygFonden til godkendelse. Det gælder dog ikke ændringer i ansøgningens projektbeskrivelse, der er foranlediget af peer review'ernes forbedringsforslag.

Udbetaling

Støtte vil blive udbetalt efter dokumentation for udgifter, medmindre særlige forhold gør sig gældende. Når du ønsker dele af eller hele bevillingen udbetalt, skal du ind på vores hjemmeside Tryghed.dk, klik på "Log in", åbne ansøgningen og følge anvisningerne.

Støtte skal udbetales senest en måned efter den slutdato, du har opgjort i ansøgningen. Er dette ikke sket, vil støtten bortfalde. Ændres slutdatoen for projektet skal dette godkendes af TrygFonden pr. mail til Pia Hansen, ph@trygfonden.dk.

Offentliggørelse

Rapporter, pjecer, videnskabelige publikationer, afhandlinger, artikler i tidsskrifter eller anden omtale af projektet skal tydeligt angive, at projektet er gennem-ført med støtte fra TrygFonden. Ønskes der sparring og eller input til formidlingen af projektet, er I velkomne til at tage kontakt til Christian Nørr, TrygFondens dokumentarist, på tlf. 25 45 62 26 eller cn@trygfonden.dk.

Desuden bedes du indsende et eksemplar til fonden, påført ID-nummer.

Evaluering

TrygFonden ønsker at samle og formidle resultaterne fra de projekter, som fonden har doneret midler til. Alle donationsmodtagere bliver derfor bedt om at udfylde et evalueringsskema. Når dit projekt nærmer sig sin afslutning, vil du modtage en mail, der indeholder et link til en evalueringsside. Du bedes evaluere dit projekt senest 14 dage efter projektets afslutning.

Regnskab

Endeligt regnskab for projektet skal indsendes til TrygFonden umiddelbart efter projektets afslutning. Regnskabet skal være underskrevet af en statsautoriseret eller registreret revisor. I tilfælde af at bevillingen kun dækker en del af det ansøgte beløb og projektets samlede budget, skal regnskabet alligevel omfatte samtlige indtægter og udgifter for projektperioden.

Vi gør opmærksom på, at den del af donationen, som evt. måtte dække

honorar/lønudgifter, er skattepligtig indkomst.

Tilbagebetaling

Bevillingen skal tilbagebetales, hvis:

• Der ikke aflægges et fyldestgørende underskrevet regnskab.

• Bevillingen eller dele heraf ikke er anvendt i overensstemmelse med betingelserne for bevillingen.

Hvis der er modtaget støtte fra anden side således, at projektet er overfinansieret, vil TrygFonden i så tilfælde kunne anmode om forholdsmæssig tilbagebetaling. I tilfælde af at der efter projektperioden er et uforbrugt beløb, skal dette tilbagebetales.

Vi ønsker held og lykke med projektet og glæder os til at høre mere om det.

Venlig hilsen

TrygFonden

Rie Odsbjerg Werner

Adm. direktør

Denne mail kan ikke besvares.

TrygFonden smba (TryghedsGruppen smba) • Hummeltoftevej 49 • DK-2830 Virum

Telefon 45 26 08 00 • Fax 45 26 0801 • info@trygfonden.dk • tryghed.dk

---

Denne e-mail indeholder fortrolig information. Hvis du ikke er den rette modtager af denne e-mail eller hvis du modtager den ved en fejltagelse, beder vi dig venligst informere afsender om fejlen ved at bruge svarfunktionen. Samtidig bedes du slette e-mailen med det samme uden at videresende eller kopiere den.
